# Medicine–Food Homology Plants and Bioactive Compounds in Polyendocrine Metabolic Ovarian Syndrome: Multi-Target Mechanisms and Functional Food Potential—A Review

**DOI:** 10.3390/nu18172837

**Published:** 2026-08-28

**Authors:** Qiuni Yang, Linzuo Zou, Yuxue Huang, Qingfeng Zhang, Chengyao He, Li Cheng, Chao Zhao

**Affiliations:** 1 The Second Clinical Medical College, Guizhou University of Traditional Chinese Medicine, Guiyang 550002, China; yangqiuni2026@126.com (Q.Y.); zoulinzuo@126.com (L.Z.); huangyuxue2026@163.com (Y.H.); qingfeng662014@126.com (Q.Z.); hechengyao2026@126.com (C.H.); 2 Yuni Township Health Centre, Panzhou 553523, China; 3 The Research Center for Quality Control of Natural Medicine, Guizhou Normal University, Guiyang 550001, China

**Keywords:** polycystic ovary syndrome, insulin resistance, hyperandrogenism, oxidative stress, inflammation, steroidogenesis, dietary intervention

## Abstract

Polyendocrine metabolic ovarian syndrome (PMOS), renamed from polycystic ovary syndrome (PCOS) by global consensus in May 2026, is one of the most common endocrine diseases in women of reproductive age, affecting physical and mental health. Growing evidence suggests that medicine–food homology (MFH) plants and their bioactive compounds may help regulate PMOS-related metabolism and reproductive abnormalities through dietary intervention. This review summarizes their reported roles and potential mechanisms. Based on the existing literature, this review presents nine edible plant resources identified under the MFH/dietary-use framework and 21 bioactive constituents or constituent classes, all selected for detailed synthesis from a broader eligible evidence base and with reported PMOS-related bioactivity. Across preclinical studies, these constituents appear to act through convergent, multi-target mechanisms, including anti-inflammatory and antioxidant activities to reduce systemic inflammation, as well as restoring hormone balance, correcting abnormal lipid metabolism, relieving insulin resistance and protecting ovarian function. Importantly, the evidence base remains predominantly preclinical, with few human randomized trials. Key translational barriers include insufficient standardization of preparations and doses, limited oral bioavailability of several constituents, safety and herb–drug interaction concerns, and uncertain formulation feasibility for functional-food applications. By integrating evidence at the plant and compound levels, this review provides a mechanistic rationale—rather than clinical proof—for further investigation of dietary strategies relevant to PMOS and proposes a framework for the development and evaluation of MFH-based functional foods.

## 1. Introduction

Polyendocrine metabolic ovarian syndrome (PMOS), previously known as polycystic ovary syndrome (PCOS), is one of the most common endocrine and metabolic disorders in women of childbearing age. In May 2026, a multistep global consensus process formally replaced the term “polycystic ovary syndrome” with “polyendocrine metabolic ovarian syndrome” [1]. The new nomenclature is used throughout this review. Diagnosis requires at least two of the following three criteria, after exclusion of related disorders: clinical or biochemical hyperandrogenism, ovulatory dysfunction, and polycystic ovarian morphology (a radiological descriptor that is unchanged by the renaming) [2]. Insulin resistance, chronic low-grade inflammation and broader metabolic disorders are not diagnostic criteria but are common associated features that affect long-term outcomes. In addition to infertility and irregular menstruation, PMOS is also associated with obesity, type 2 diabetes, increased cardiovascular risk and significant psychological burden, which has a profound impact on long-term health and socioeconomic outcomes. Globally, PMOS affects approximately one in eight women [1,3]. The current first-line treatment includes metformin and other insulin sensitizers, ovulation induction agents such as clomiphene citrate or letrozole, and combined oral contraceptives. However, each program has its own limitations: ovulation-promoting drugs have the risk of clomiphene resistance and multiple pregnancy; combined oral contraceptives are unsuitable for those with fertility needs; and long-term pharmacotherapy is limited by side effects and compliance instability. These limitations, combined with the growing preference of patients for dietary and lifestyle interventions, have prompted the search for safe and sustainable adjunct strategies.

Because the nomenclature change occurred only recently, clarification of the terminology used in the evidence base is necessary. All primary studies included in this review were published before May 2026 and therefore used the term PCOS to describe the condition, study populations, diagnostic criteria, and experimental models. These populations, models, and pathophysiological processes correspond to the same clinical entity now designated as PMOS. To preserve bibliographic accuracy and methodological reproducibility, the original term PCOS is retained in the titles of cited publications, database search strategies, and the formal names of organizations, guidelines, and validated instruments. PMOS is otherwise used consistently throughout the main text, tables, figures, and abbreviation list. Accordingly, references to PMOS in this review encompass evidence originally reported in the literature under the term PCOS.

PMOS is a chronic disease that requires lifelong management. Many of its core pathological characteristics—insulin resistance, chronic low-grade inflammation and oxidative stress—are themselves responsive to dietary intervention. Against this background, dietary strategies and functional foods have become an important complementary approach for controlling symptoms and supporting long-term metabolic health. In traditional East Asian dietary practice, medicine–food homology (MFH) plants have long served both nutritional and medicinal functions and remain part of the daily diet [4]. These plants are rich in bioactive compounds such as flavonoids, phenolic acids, terpenes, saponins, isoflavones and amino acid derivatives. Preclinical pharmacological evidence shows that these ingredients can not only enhance insulin sensitivity and relieve oxidative–inflammatory stress but also regulate androgen biosynthesis and support follicular development in the PMOS models [5,6].

Despite this preclinical promise, the existing literature often treats these plants as medicinal extracts or natural drugs. Their edible properties, and their potential to be developed into functional foods that target systemic inflammation and metabolic disorders, are largely overlooked. Accumulating in vitro and in vivo evidence supports their potential biological relevance to PMOS-related metabolic and reproductive pathways. Even so, systematic and integrative research from a food perspective is still lacking—research that links edible plant sources, key bioactive compounds, underlying mechanisms, and the feasibility of functional-food development.

Reviews of natural products and dietary interventions in PMOS are already available, but they differ from the present review in scope and evidence organization. Yuan et al. [7] focused on chemically defined natural compounds and their effects on hormonal regulation, explicitly excluding complex multi-component formulations. Muhammed Saeed et al. [8] reviewed dietary patterns, macronutrients, micronutrients, dietary supplements, and herbal interventions within a broad nutritional-management framework. Lee et al. [9] surveyed natural remedies, including herbs, herbal formulations, and acupuncture, across preclinical, mechanistic, and clinical evidence, whereas Wang et al. [10] synthesized human evidence on dietary supplements using an umbrella meta-analytic approach. In contrast, the present review uses MFH status or documented edible use as an entry criterion and explicitly separates plant/preparation-level evidence from chemically defined compound-level evidence. It also distinguishes effects demonstrated with experimentally tested extracts, essential oils, enriched fractions, or isolated compounds from those that can reasonably be extrapolated to habitual dietary intake. Building on this evidence structure, the review further addresses preparation standardization, bioavailability, safety, herb–drug interactions, and formulation issues relevant to the development of MFH-based functional foods.

This review aims to fill this gap. Specifically, it sets out to (i) summarize commonly used MFH plants and their main bioactive constituents; (ii) review the evidence for their anti-PMOS effects from cell-based, animal, and available clinical studies; and (iii) clarify the mechanistic pathways through which they regulate metabolism, inflammation, oxidative stress, and reproductive endocrine balance. By integrating evidence at both the plant and compound levels and clearly distinguishing between preclinical and clinical findings, this review aims to provide a structured and critically evaluated basis for functional-food development and clinical nutrition research in PMOS.

## 2. Materials and Methods

This study is a narrative review with a structured literature screening and criteria-based evidence-selection strategy. The literature retrieval database includes PubMed, Web of Science and CNKI. The retrieval time range is from database inception to December 2025. Search terms were used alone or in combination, including: “polycystic ovary syndrome”, “PCOS”, “medicine–food homology”, “medicinal and edible”, “food–medicine”, “functional food”, “edible medicinal plant”, “bioactive compound”, “phytochemical”, “insulin resistance”, “hyperandrogenism”, “oxidative stress”, “inflammation”, “ovarian function” and “steroidogenesis”. The terms “polycystic ovary syndrome” and “PCOS” were retained as search strings because they were the indexing terms in use in all bibliographic databases throughout the retrieval period; the corresponding condition is referred to as PMOS throughout this review.

The literature search was conducted and updated on 31 December 2025. This date was considered the final search date for the study-selection process, and eligibility for the evidence synthesis was restricted to studies published on or before this date. The exact search strategies were as follows. Additional searches were performed using the botanical names of candidate plants and the names of their major constituents. Reference lists of relevant articles were also manually checked to identify studies that may have been missed. Publications in English and Chinese were considered. Priority was given to peer-reviewed original studies, randomized controlled trials, animal experiments, mechanistic cell studies and recent reviews related to PMOS, medicine–food homology resources, edible medicinal plants, functional foods and food-derived bioactive compounds.

The PubMed search was performed as follows: (“Polycystic Ovary Syndrome”[MeSH] OR “polycystic ovary syndrome”[tiab] OR PCOS[tiab]) AND (“medicine food homology”[tiab] OR “medicinal and edible”[tiab] OR “food-medicine”[tiab] OR “functional food”[tiab] OR “edible medicinal plant”[tiab] OR “bioactive compound*”[tiab] OR phytochemical*[tiab] OR flavonoid*[tiab] OR polysaccharide*[tiab] OR alkaloid*[tiab] OR terpenoid*[tiab] OR saponin*[tiab]) AND (“insulin resistance”[tiab] OR hyperandrogenism[tiab] OR “oxidative stress”[tiab] OR inflammation[tiab] OR “ovarian function”[tiab] OR steroidogenesis[tiab]). Filters: English, Chinese; database inception to 31 December 2025. The Web of Science (Core Collection) search was performed as follows: TS=(“polycystic ovary syndrome” OR PCOS) AND TS=(“medicine food homology” OR “medicinal and edible” OR “functional food” OR “edible medicinal plant” OR “bioactive compound*” OR phytochemical* OR flavonoid* OR polysaccharide* OR alkaloid* OR terpenoid* OR saponin*) AND TS=(“insulin resistance” OR hyperandrogen* OR “oxidative stress” OR inflammation OR “ovarian function” OR steroidogenesis). Timespan: inception to 31 December 2025; languages: English, Chinese. The CNKI (China National Knowledge Infrastructure) search was performed as follows: SU=(‘多囊卵巢综合征’ + ‘PCOS’) AND SU=(‘药食同源’ + ‘药食两用’ + ‘功能性食品’ + ‘活性成分’ + ‘黄酮’ + ‘多糖’ + ‘生物碱’ + ‘葛根’ + ‘葛属’ + ‘葛根素’ + ‘皂苷’) AND SU=(‘胰岛素抵抗’ + ‘高雄激素血症’ + ‘氧化应激’ + ‘炎症’ + ‘卵巢功能’). Coverage: inception to 31 December 2025 (“+” denotes OR; SU = subject field).

Plant-level resources were considered eligible when they met both of the following criteria: (i) the plant is listed in the Chinese catalogue of substances traditionally regarded as both food and Chinese medicinal materials (the “medicine–food homology” catalogue), which was established through the 2002 List of Items That Are Both Food and Drugs and subsequently expanded through official announcements in 2019, 2023, and 2024, comprising 106 substances at the time of writing [11,12,13,14], or, where the evaluated species or plant part is not covered by that catalogue, has a documented history of daily dietary, culinary, or beverage use, with the botanical source and plant part traceable; and (ii) at least one peer-reviewed study directly evaluated the plant material itself or a multi-constituent preparation, such as an extract, essential oil, or enriched fraction, in a recognized PMOS-related in vivo, in vitro, or human setting. Evidence derived solely from an isolated, chemically defined constituent was assigned to the compound-level synthesis in Section 4 rather than to the plant-level synthesis in Section 3. The regulatory framework applied in this review is that of mainland China and is governed by the Administrative Provisions for the Catalogue of Substances Traditionally Regarded as Both Food and Chinese Medicinal Materials [15]. Medicine–food homology status is jurisdiction-specific, and inclusion here does not imply that a given resource is approved as a food ingredient in other regulatory systems. Among the included resources, most correspond to materials listed in the Chinese catalogue, although catalogue status is plant-part specific. For example, *Crocus sativus* stigma, *Curcuma longa* rhizome, and *Angelica sinensis* root are listed specifically for use as spices and seasonings [12], whereas other resources, such as Pueraria tuberosa, qualified for inclusion in this review on the basis of documented dietary use rather than formal catalogue listing; the regulatory or dietary-use basis for each plant is indicated in Table 1. Eligible PMOS-related outcomes included changes in reproductive-hormone profiles, ovarian morphology or histology, estrous or menstrual cyclicity, insulin resistance, glucose or lipid metabolism, inflammatory or oxidative-stress markers, and other measures of ovarian function. Among eligible plant-level resources, priority was given to those that added non-redundant coverage of the PMOS-related outcome domains and preparation types represented in the retrieved literature.

Isolated compounds were considered for inclusion when their chemical structure was clear and PMOS-related evidence was available. Acceptable study systems included established rodent PMOS models using hormonal and/or metabolic induction paradigms (e.g., letrozole, dehydroepiandrosterone, testosterone, estradiol valerate, or combined high-fat-diet protocols), other established PMOS induction paradigms, PMOS-relevant ovarian-cell systems such as KGN granulosa-like cells, and studies in women with PMOS. PMOS-related computational studies were retained only when explicitly identified as hypothesis-generating evidence and were not treated as equivalent to direct in vivo, in vitro, or human evidence. Compounds with unclear chemical identity, lacking PMOS-related evidence, or supported only by non-PMOS evidence such as general metabolic regulation or antioxidant activity were not included in the main review. Among the compounds meeting these criteria, twenty-one were selected for detailed synthesis based on the relevance and directness of the available PMOS-specific evidence. The compound-level synthesis was not restricted to constituents of the nine plant-level resources selected for Section 3; compounds meeting the compound-level criteria could therefore be included even when their reported plant/food sources were not represented in Section 3. To facilitate comparison across resources, Tables 1 and 2 include a descriptive evidence profile based on the PMOS-related studies summarized in this review. The profile distinguishes human-participant, animal, cellular, mechanism-focused, and computational evidence and is intended to describe evidence domains and mechanistic directness rather than to provide a formal certainty-of-evidence grade.

Accordingly, the nine plant resources presented in Section 3 and the twenty-one compounds presented in Section 4 represent selected resources for detailed narrative synthesis rather than an exhaustive inventory of all resources eligible under the MFH/dietary-use framework.

Title/abstract screening and full-text eligibility assessment were conducted by one author, and the screening decisions and final eligibility assessments were subsequently verified by a second author. Thus, study selection involved secondary verification rather than independent duplicate screening. To reduce subjective selection, eligibility was assessed against the explicit plant- and compound-level criteria described above, and selection for detailed synthesis followed the stated criteria for non-redundant coverage and the relevance and directness of PMOS-specific evidence. Neither outcome direction nor statistical significance was used as an eligibility or prioritization criterion. Within the included evidence, non-significant or divergent findings were retained and reported where present rather than excluded on the basis of outcome direction or significance and were interpreted in the context of differences in study design, experimental model, preparation, and dose. Because this review is narrative and the detailed resource sets are non-exhaustive, residual selection bias cannot be excluded. The literature search identified 699 records (PubMed, 289; Web of Science, 233; CNKI, 177). After removal of 181 duplicates, 518 records were screened by title and abstract, of which 397 were excluded as off-topic. The remaining 121 full-text articles were assessed against the eligibility criteria above; 63 were excluded (27 with no PMOS-relevant outcome or not an intervention; 21 evaluating a compound not derived from an MFH/edible plant or of unclear chemical identity; and 15 reviews, duplicate datasets, or records without a retrievable full text). Fifty-eight PMOS-related primary studies—evaluating 9 plant-level resources and 21 chemically defined bioactive compounds—were included in the narrative synthesis. Because this is a narrative rather than a systematic review, additional references providing physiological, phytochemical, mechanistic, safety, or methodological context are cited throughout the text; these background sources are distinct from, and not counted among, the 58 included primary studies. Selected references published after the final search date were cited only to provide updated contextual information and were not included in the study-selection process or the 58-study evidence synthesis.

**Scheme 1:**
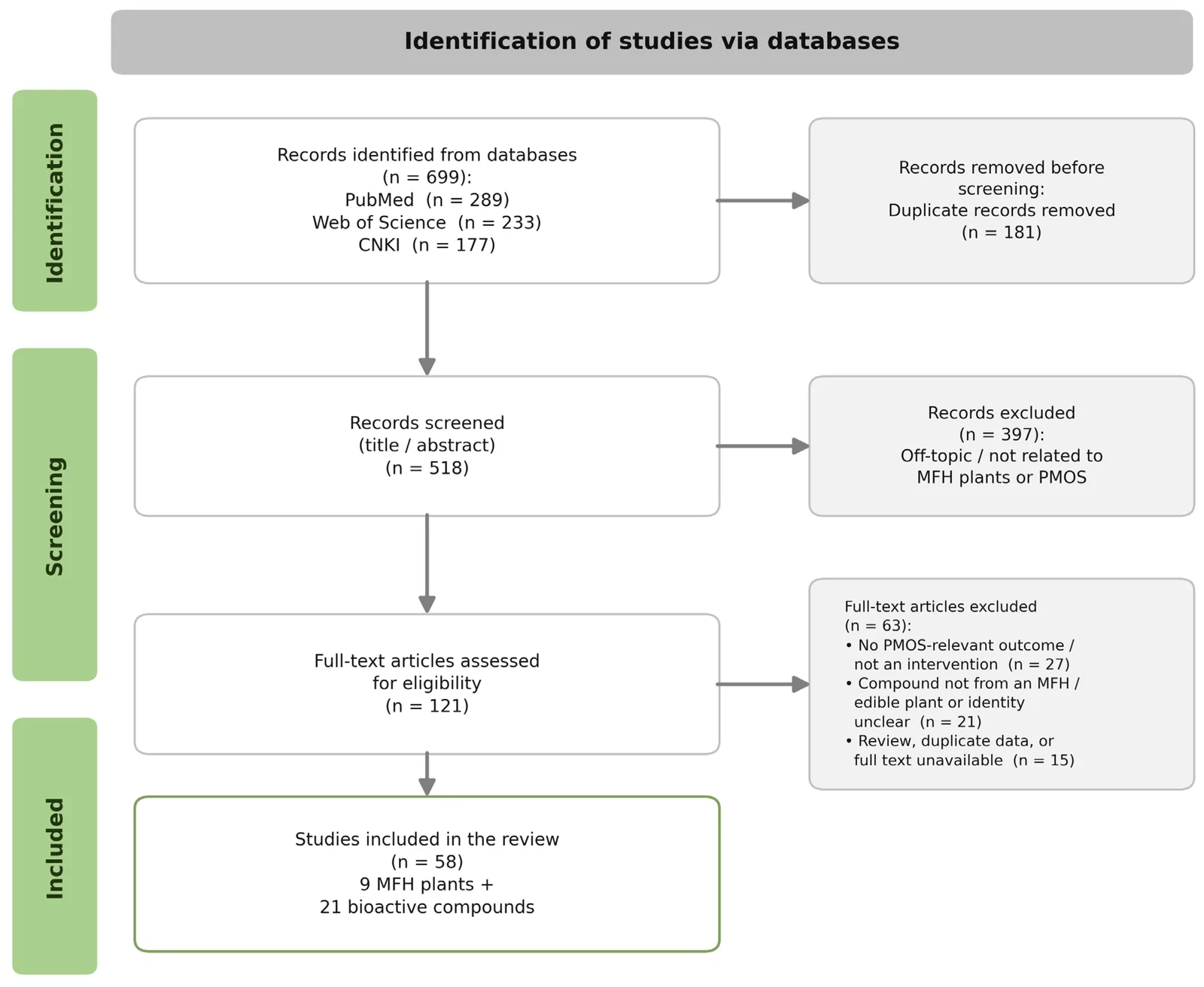
PRISMA-informed flow diagram of the literature identification and study-selection process for this narrative review, showing the identification, screening, and eligibility assessment of the retrieved records. The database indexing term in use throughout the retrieval period (inception to 31 December 2025) was “polycystic ovary syndrome”/“PCOS”, which was therefore used as the search string; the corresponding condition is referred to as PMOS throughout this review. The final search was conducted and updated on 31 December 2025, and the evidence synthesis was restricted to studies published on or before that date. Fifty-eight PMOS-related primary studies informing the nine plant-level resources and twenty-one compound-level resources presented in Section 3 and Section 4 were included in the narrative synthesis. These resource counts reflect the evidence-level framework and criteria-based selection described in Section 2 and should not be interpreted as an exhaustive inventory of all resources eligible under the MFH/dietary-use framework. Other cited references are background/contextual sources and are not counted among the included studies. MFH, medicine–food homology; PMOS, polyendocrine metabolic ovarian syndrome. Adapted from PRISMA 2020 (https://www.prisma-statement.org/prisma-2020-flow-diagram; accessed on 31 December 2025).

In view of the wide scope of this topic and the heterogeneity of existing evidence in plant sources, isolated compounds, experimental models, preparation forms, doses and outcome measures, narrative synthesis was more suitable for this article than a quantitative systematic review or meta-analysis. Human research is retained in the full text and table and is clearly distinguished from animal and in vitro research. This article reports on the retrieval database, the exact search strings and the final search date (31 December 2025), the retrieval scope, language restrictions, the inclusion criteria, the study-selection counts summarized in the PRISMA-style flow diagram (Scheme 1), and the evidence-classification methods to improve methodological transparency and reproducibility while reflecting the characteristics of a narrative review with a structured, criteria-based selection. The flow diagram is presented for the transparency of literature identification and screening rather than as a systematic review process. This review is framed as a medicine–food homology and functional-food resource review rather than a clinical-efficacy review. Accordingly, the evidence synthesis is predominantly preclinical: among the 58 included primary studies, 55 are animal or in vitro studies, whereas only three are human randomized controlled trials. These three trials are retained solely to provide limited translational context and to delineate the current boundary of human evidence; they are discussed separately in Section 4.7 and are not used to support conclusions regarding clinical efficacy.

## 3. Edible Plant Sources Used for PMOS

Given their established or documented dietary use and reported multi-target biological activities, MFH plants have attracted increasing attention in PMOS-related dietary research. Preclinical studies and limited human research suggest that these plants may modulate multiple PMOS-related metabolic, inflammatory, and reproductive pathways. The following section presents nine representative plant-level resources selected according to the criteria described in Section 2, focusing on their dietary uses, main bioactive constituents, and reported anti-PMOS effects (Table 1).

### 3.1. Fennel

Fennel (*Foeniculum vulgare* Mill.) is an Apiaceae plant widely used as a culinary spice. Its fruits and seeds contain volatile oils, flavonoids, phenolic compounds, and terpenoids, which provide a phytochemical basis for its reported endocrine and other biological activities [16]. In an estradiol-valerate-induced PMOS model in female Wistar rats, treatment with fennel essential oil for 14 days significantly increased serum estrogen and progesterone levels and reduced the number of ovarian cysts compared with untreated PMOS animals [17]. Although oxidative stress is an important component of PMOS pathophysiology and contributes to ovarian dysfunction [18], the cited PMOS study did not demonstrate a significant reduction in malondialdehyde levels following fennel-essential-oil treatment [17]. Separately, a methanolic extract of *F. vulgare* fruit demonstrated antioxidant and anti-inflammatory activities in non-PMOS experimental models, increasing plasma SOD and catalase activities and reducing lipid peroxidation, as reflected by lower malondialdehyde levels [19]. Given its established culinary use, fennel remains of interest from a food perspective. However, the available PMOS evidence is based on fennel essential oil rather than ordinary dietary use, and it is not yet clear whether food-based preparations would provide a comparable exposure.

### 3.2. Licorice

Licorice is derived from the dried roots and rhizomes of *Glycyrrhiza uralensis Fisch.* or *Glycyrrhiza glabra* L. It is utilized as a natural sweetener in various confectionery and beverages. It contains triterpenes, flavonoids and polysaccharides, which can promote anti-inflammatory effects and metabolic regulation. In a randomized, double-blind, placebo-controlled trial [20], 66 overweight or obese women with PMOS (33 per group) received licorice extract (1.5 g/day) plus a low-calorie diet or placebo plus the same diet for 8 weeks. The licorice group showed reductions in body weight (88.84 to 82.60 kg; −6.24 kg, −7.0%), BMI (30.67 to 29.03 kg/m^2^), body-fat percentage (30.66% to 28.21%), and fasting blood glucose (100.03 to 74.00 mg/dL; −26.0%). Lipid parameters also improved, with reductions in triglycerides, total cholesterol, and LDL-C (−37.2%, −23.5%, and −33.0%, respectively) and an increase in HDL-C (+12.2%) (all *p* < 0.05). However, because both groups received a low-calorie diet, these effects cannot be attributed solely to licorice. The extract was reported to contain 7.03% glycyrrhizic acid (reported as 36.5 mg per 500 mg capsule), which should be considered in safety evaluation. Animal studies further showed reduced testosterone levels and improved ovarian morphology in PMOS models [21]. These findings support further evaluation of standardized licorice preparations for PMOS-related metabolic and endocrine outcomes; however, the limited human evidence and glycyrrhizic acid-related safety considerations preclude firm functional-food or clinical recommendations.

### 3.3. Pueraria tuberosa

*Pueraria tuberosa* (Roxb. ex Willd.) DC., commonly known as Indian kudzu or vidari kand, is a tuberous legume with documented traditional nutritional and medicinal uses. Its tuber has traditionally been used in milk-based preparations and in formulations containing wheat or barley, ghee, and milk, and it contains several isoflavones, including puerarin, daidzein, and genistein [22]. In a letrozole-induced PMOS rat model, *P. tuberosa* intervention improved the sex-hormone profile and estrous cyclicity [23]. In addition, a *P. tuberosa* water extract exhibited antioxidant activity and reduced serum triglyceride and total cholesterol levels in a high-fat-diet-induced model of hepatic steatosis [24]. Taken together, these findings make *P. tuberosa* relevant to PMOS-related food research, but the evidence should be interpreted cautiously. The direct PMOS study used an ethanolic tuber extract, and it is not known whether traditional food preparations would result in similar exposure. In addition, the lipid-modulating and antioxidant findings come partly from non-PMOS models and therefore provide only supportive context.

### 3.4. Eucommia Folium

*Eucommia ulmoides* Oliv. (*E. Folium*) dried leaves are widely used as herbal tea in China and as a functional ingredient for soups and porridge. The leaves are rich in flavonoids, phenolic acids and lignans, which have antihypertensive, lipid-lowering, antioxidant and neuroprotective activities [25]. In vitro and animal studies have confirmed the potential of *E. Folium* extract in alleviating PMOS and related metabolic disorders. In a rat insulin-resistant PMOS (PMOS-IR) model, 21-day intervention can effectively inhibit abnormal ovarian morphology and alleviate insulin resistance and sex-hormone disorders [26]. Specifically, the extract increases the levels of serum follicle-stimulating hormone (FSH), estradiol (E2) and progesterone (P), while reducing the concentration of luteinizing hormone (LH), testosterone (T) and insulin (INS) [27]. These results suggest its regulatory effect on the hypothalamic–pituitary–ovarian (HPO) axis and insulin signaling pathway. It is worth noting that the same extract has also been proven to correct gut dysbiosis associated with hyperlipidemia [28] —a process that is increasingly considered to be related to the metabolism and androgen drive of PMOS—suggesting a complementary intestinal–endocrine pathway. Overall, the findings support the continued study of *E. Folium* in the PMOS setting. The evidence, however, comes from concentrated flavonoid preparations, so it cannot yet be assumed that herbal tea or other usual food preparations would deliver a comparable exposure or produce the same effects.

### 3.5. Saffron

Saffron is derived from the dried stigma of *Crocus sativus* L. and is widely used worldwide as a food flavoring and coloring agent. The stigma is characterized by carotenoids such as crocin and crocetin and by the monoterpene aldehyde safranal, whereas the petals contain a different phytochemical profile enriched in anthocyanins, flavonoids, and other phenolic compounds [29]. In a testosterone-induced PMOS mouse model, a *C. sativus* petal extract and an anthocyanin preparation reduced serum LH, testosterone, and E2 levels while restoring FSH and progesterone towards normal levels [30]. Gene-expression analysis further showed that the petal-derived interventions upregulated gonadotropin receptors (*Fshr* and *Lhr*) and steroid-hormone receptors (*Pgr* and *Esr1*), reduced the inflammatory markers TNF-α and IL-6, and enhanced the activities of the antioxidant enzymes GPx, SOD, CAT, and GST. These findings support further investigation of *C. sativus*-derived preparations in PMOS while emphasizing that the edible stigma spice and petal-derived preparations are chemically distinct materials and should not be treated as interchangeable.

### 3.6. Turmeric

The rhizome of *Curcuma longa* L. (Turmeric) is a common dietary spice. Its main biologically active ingredients include turmeric and volatile oils, which have strong anti-inflammatory and antioxidant activities directly related to PMOS [31]. In a letrozole-induced PMOS model, it was shown that taking an oral turmeric extract for 30 days can reshape the hormone secretion pattern and improve the lipid profile. Mechanism studies show that the extract restores the estrous cycle by improving ovarian morphology and alleviating cell dysfunction [32]. The turmeric extract showed activity across several PMOS-related outcomes in the preclinical model. Whether these effects are relevant to the much lower exposure associated with ordinary culinary use of turmeric remains uncertain.

### 3.7. Lycium barbarum Leaf

The leaves of *Lycium barbarum* L. have been used as leafy vegetables and herbal teas in China for a long time [33]; they are rich in polysaccharides, flavonoids and terpenes and have strong antioxidant and glucose–lipid regulatory effects [34]. In a PMOS mouse model, a 29-day *L. barbarum* leaf extract intervention improved the circulating reproductive-hormone spectrum, reduced ovarian histopathological changes, and partially restored the estrous cycle [35]. Because *L. barbarum* leaves are traditionally consumed as vegetables, they warrant further evaluation as an edible plant source for PMOS-related dietary research; however, the effects observed with the tested extract should not be assumed to occur with habitual leaf consumption.

### 3.8. Raspberry

Raspberry (*Rubus chingii* Hu) can be eaten fresh or made into traditional dry products, and it is rich in terpenoids, alkaloids, flavonoids and fatty acids. These bioactive ingredients have been reported to have antioxidant, anti-inflammatory, hypolipidemic, hepatoprotective and anti-cancer properties [36]. Recent studies [37] have indicated that extracts of *R. chingii* may exert beneficial effects in PMOS animal models. Regarding reproductive phenotypic improvements, raspberry extracts have been associated with reduced follicular atresia, enhanced ovulatory function, and improved ovarian histopathology. Furthermore, they appear to modulate sex-hormone dysregulation and contribute to metabolic homeostasis, which may help mitigate the adverse effects of metabolic disturbances on the reproductive system. These findings justify further study of R. chingii preparations in PMOS, but the experimentally tested preparation differs substantially from ordinary fresh or dried fruit consumption, so comparable effects should not be assumed.

### 3.9. Angelica sinensis

*Angelica sinensis* Radix is a culinary herb routinely added to soups, stews, and herbal teas across East Asia. Its biological actions are primarily attributed to Angelica polysaccharides, organic acids, and volatile oils, which have been reported to exert immunomodulatory, anti-inflammatory, anti-apoptotic, and anticancer effects [38]. Multiple in vivo experiments have shown that *A. sinensis* extracts (aqueous or ethanolic) may ameliorate the pathological progression of PMOS. These extracts have been associated with the attenuation of serum hyperandrogenemia, improvement in estrous-cycle irregularities, and amelioration of insulin resistance and dyslipidemia [39,40]. Furthermore, the extracts mitigate PMOS-related chronic inflammation, inhibit inflammatory interaction with the reproductive axis, and reduce ovarian tissue lesions. The primary proposed mechanism involves reducing ovarian oxidative stress and enhancing systemic antioxidant capacity, thereby protecting the follicular microenvironment [41,42]. Its long-standing culinary use makes *A. sinensis* relevant to functional-food research; however, the reported PMOS-related effects derive from specific aqueous or ethanolic extracts and require further translational evaluation.

An important limitation of the plant-level evidence summarized in this section is that most PMOS-related studies evaluated extracts, essential oils, enriched fractions, or other multi-constituent preparations rather than the corresponding edible plant material under habitual dietary conditions. In some cases, the experimentally evaluated preparation also differs from the customary edible plant part, as illustrated by the petal-derived preparation of Crocus sativus evaluated in the PMOS study, whereas the stigma is the part conventionally consumed as saffron. Because the qualitative and quantitative composition of such preparations may vary with botanical source, plant part, extraction solvent, processing procedure, batch, and dose, the observed effects cannot be attributed to an individual constituent unless that constituent was directly evaluated, nor can they be assumed to occur after ordinary dietary intake of the corresponding edible material. Accordingly, the findings in Section 3 are interpreted as effects of the specific preparation and dose tested. The listed bioactive constituents describe the known phytochemical profile of each plant or preparation and are not necessarily the causal mediators of the reported PMOS-related effects. To avoid conflating evidence levels, plant- and preparation-level evidence is discussed separately from evidence for chemically defined isolated compounds in Section 4.

Taken together, the studies of these nine edible plant sources indicate that the tested preparations may influence multiple PMOS-related endpoints rather than a single pathological process. Across the preparations evaluated, reported effects span endocrine-reproductive, metabolic, inflammatory, and oxidative-stress domains, although the strength and relevance of the evidence vary substantially according to the preparation and experimental model used. At the endocrine-reproductive level, several preparations were associated with changes in reproductive-hormone profiles, ovarian morphology, and follicular or estrous-cycle outcomes. Metabolic outcomes reported across the studies included improvements in insulin resistance and lipid-related parameters, while some preparations were also associated with changes in inflammatory and oxidative-stress markers. However, the relative contribution of these pathways varies among preparations and models. These findings therefore provide a rationale for further standardized preclinical and translational evaluation rather than evidence for established dietary efficacy in PMOS management.

Evidence strength is nevertheless uneven across the nine plant-level resources. Licorice is the only plant-level resource represented by a human randomized trial, but the concurrent low-calorie diet limits attribution of the observed effects to licorice alone [20]. Most other resources are supported primarily by one or a small number of rodent studies. Additional limitations include supportive metabolic evidence derived from a non-PMOS model for Pueraria tuberosa [24] and the mismatch between the Crocus sativus petal preparation evaluated in the PMOS study and the stigma, conventionally consumed as saffron [30]. Thus, convergence in reported outcomes should not be interpreted as equivalent evidence quality across plant resources.

**Table 1:** Edible plant resources, their MFH inclusion basis/regulatory status in mainland China, reported PMOS-related effects, and comparative evidence profiles.

Botanical Name	MFH Inclusion Basis/Regulatory Status in Mainland China	Edible Form/Preparation Evaluated	Reported Principal Bioactive Constituents	Reported PMOS-Related Effects and Mechanisms	PMOS Model/Study System	Evidence Profile	Direct PMOS-Related Evidence Reference(s)
*Foeniculum vulgare* Mill. (fennel)	2002 catalogue (fennel) [11]	Culinary spice; fennel essential oil	Flavonoids, phenols, terpenoids	Restoration of hormonal balance, reflected by increased estrogen and progesterone; reduction in ovarian cyst number and improvement in ovarian histology	Estradiol-valerate-induced PMOS model in female Wistar rats	A	[17]
*Glycyrrhiza* spp. (licorice)	2002 catalogue (licorice) [11]	Sweetener/functional-food ingredient; licorice extract (1.5 g/day in the human RCT) and extract evaluated in the animal study	Triterpenoids, flavonoids, polysaccharides	Reduction in BMI, fasting glucose, and serum testosterone; restoration of ovarian morphology	Estradiol-valerate-induced NMRI mouse model; randomized controlled trial in women with PMOS (human RCT + animal)	H + A	[20,21]
*Pueraria tuberosa* (Roxb. ex Willd.) DC.	Documented dietary use [22]; not treated as formally catalogue-listed in this review	Traditional tuber preparation; tuber ethanolic extract	Isoflavones (puerarin, daidzein, genistein) and flavonoids	Improved sex-hormone profile and ovarian histomorphology; reduced cystic follicles	Letrozole-induced rat model (animal)	A	[23]
*Eucommia ulmoides* Oliv. (*Eucommia Folium*)	Catalogue-listed as Eucommia leaf, 2023 announcement [13]	Herbal tea/functional ingredient; total flavonoid fraction from leaves	Flavonoids, phenolic acids, and lignans	Improvement in insulin resistance; modulation of reproductive-hormone profiles; attenuation of ovarian morphological abnormalities	Letrozole combined with high-fat-diet-induced insulin-resistant rat model; DHEA-induced rat model (animal)	A	[26,27]
*Crocus sativus* L. (saffron)	Stigma catalogue-listed; spice/seasoning use only (2019 announcement) [12]; tested petal preparation differs from listed part	Stigma used as a culinary spice/colorant; PMOS-tested preparation: petal extract and anthocyanin preparation	Stigma: carotenoids (crocin and crocetin) and safranal; petals: anthocyanins, flavonoids, and other phenolic compounds	Upregulation of gonadotropin receptors (*Fshr*, *Lhr*) and steroid-hormone receptors (*Pgr*, *Esr1*); suppression of inflammatory markers (TNF-α, IL-6); enhancement of antioxidant enzyme activity (GPx, SOD, CAT, GST); restoration of sex-hormone balance	Testosterone-enanthate-induced PMOS mouse model (animal)	A + Massoc	[30]
*Curcuma longa* L. (turmeric)	Rhizome catalogue-listed, spice use only, 2019 announcement [12]	Culinary spice; turmeric rhizome extract	Curcuminoids and volatile oils	Improved reproductive-hormone and glucose–lipid profiles; reduced oxidative/inflammatory stress; increased circulating adiponectin	Letrozole-induced mice model (animal)	A + Massoc	[32]
*Lycium barbarum* L.	Leaf included by documented dietary use [33]; 2002 catalogue entry refers to the fruit, not the tested leaf [11]	Leafy vegetable/herbal tea; aqueous leaf extract	Polysaccharides, flavonoids, and terpenoids	Restoration of circulating reproductive hormones; amelioration of ovarian histological lesions; partial recovery of estrous cyclicity	Letrozole + high-fat-diet-induced PMOS mouse model (animal)	A + Massoc	[35]
*Rubus chingii* Hu (Raspberry)	2002 catalogue (raspberry) [11]	Fresh/dried fruit; raspberry fruit extract	Terpenoids, alkaloids, flavonoids, and fatty acids	Maintenance of metabolic homeostasis; correction of sex-hormone imbalance; reduction in follicular atresia; improvement in ovulatory function and ovarian histopathology	DHEA-induced PMOS-IR rat model (animal)	A + Massoc	[37]
*Angelica sinensis* (Oliv.) Diels	Root catalogue-listed, spice use only, 2019 announcement [12]	Soup/stew ingredient; aqueous and ethanolic root extracts	*Angelica* polysaccharides, organic acids, and volatile oils	Reduction in serum androgens; restoration of estrous cyclicity; improvement in insulin resistance and dyslipidemia; suppression of ovarian oxidative stress and systemic inflammation, with consequent preservation of the follicular microenvironment	Letrozole combined with high-fat-diet-induced rat model; letrozole-induced mouse model (animal)	A + Massoc	[39,40,41,42]

Note: Regulatory status refers to the mainland China medicine–food catalogue framework established by the 2002 Ministry of Health list and subsequent official announcements [11,12,13,14,15]. Catalogue status is jurisdiction-specific and plant-part-specific. “Documented dietary use” is an inclusion basis used in this review and does not denote formal catalogue listing in mainland China. “Edible form/preparation evaluated” distinguishes customary dietary use from the specific preparation evaluated experimentally. References supporting the MFH inclusion basis/regulatory status are provided in the corresponding table column. For studies using extracts, essential oils, enriched fractions, or other multi-constituent preparations, the reported effects refer only to the specific preparation and dose tested and should not be assumed to represent equivalent effects of the corresponding whole edible plant. “Reported principal bioactive constituents” describes the known phytochemical composition of the plant or preparation; these constituents should not be interpreted as demonstrated causal mediators of the reported effects unless they were directly evaluated. “PMOS model/study system” specifies the disease-induction method or study context used in the corresponding direct evidence. Different PMOS models reproduce partially distinct endocrine, ovarian, metabolic, and inflammatory phenotypes; therefore, findings should be interpreted within the context of the specific model used and should not be assumed to be directly interchangeable across models or extrapolated to clinical efficacy. “Direct PMOS-related evidence reference(s)” includes only studies that directly evaluated the listed preparation in a PMOS-related human or animal setting. References concerning botanical identity, dietary use, phytochemistry, reviews, general pharmacology, or non-PMOS experimental models are retained only as contextual or mechanistic background in the narrative text. “Evidence profile” provides a descriptive summary of the PMOS-related evidence available for each resource: H, human-participant clinical/intervention evidence; A, in vivo animal evidence; C, cellular evidence; Mpert, mechanism-focused evidence that includes direct experimental manipulation of a proposed mediator or pathway; Massoc, mechanism-focused molecular, biochemical, signaling, omics, or microbiome evidence without direct causal perturbation; and Comp, computational hypothesis-generating evidence. Where Mpert is assigned, Massoc is not additionally listed. Mpert indicates that at least one mechanism-related experiment involved direct perturbation and does not imply that all proposed mechanisms for that resource have been causally verified. Non-PMOS general pharmacological studies are not counted in the evidence profile. The profile is descriptive and does not constitute a formal GRADE or risk-of-bias assessment. Abbreviations: BMI—body mass index; CAT—catalase; GPx—glutathione peroxidase; GST—glutathione S-transferase; IL-6—interleukin-6; MFH—medicine–food homology; PMOS—polyendocrine metabolic ovarian syndrome; RCT—randomized controlled trial; SOD—superoxide dismutase; TNF-α—tumor necrosis factor-alpha. “Animal study”—in vivo rodent studies only; “Animal + in vitro”—studies conducted in both rodent models and cell-based assays; “Human RCT + animal”—at least one published randomized controlled trial in women with PMOS alongside animal data.

## 4. Bioactive Components Responsible for PMOS Improvement

**Figure 1:**
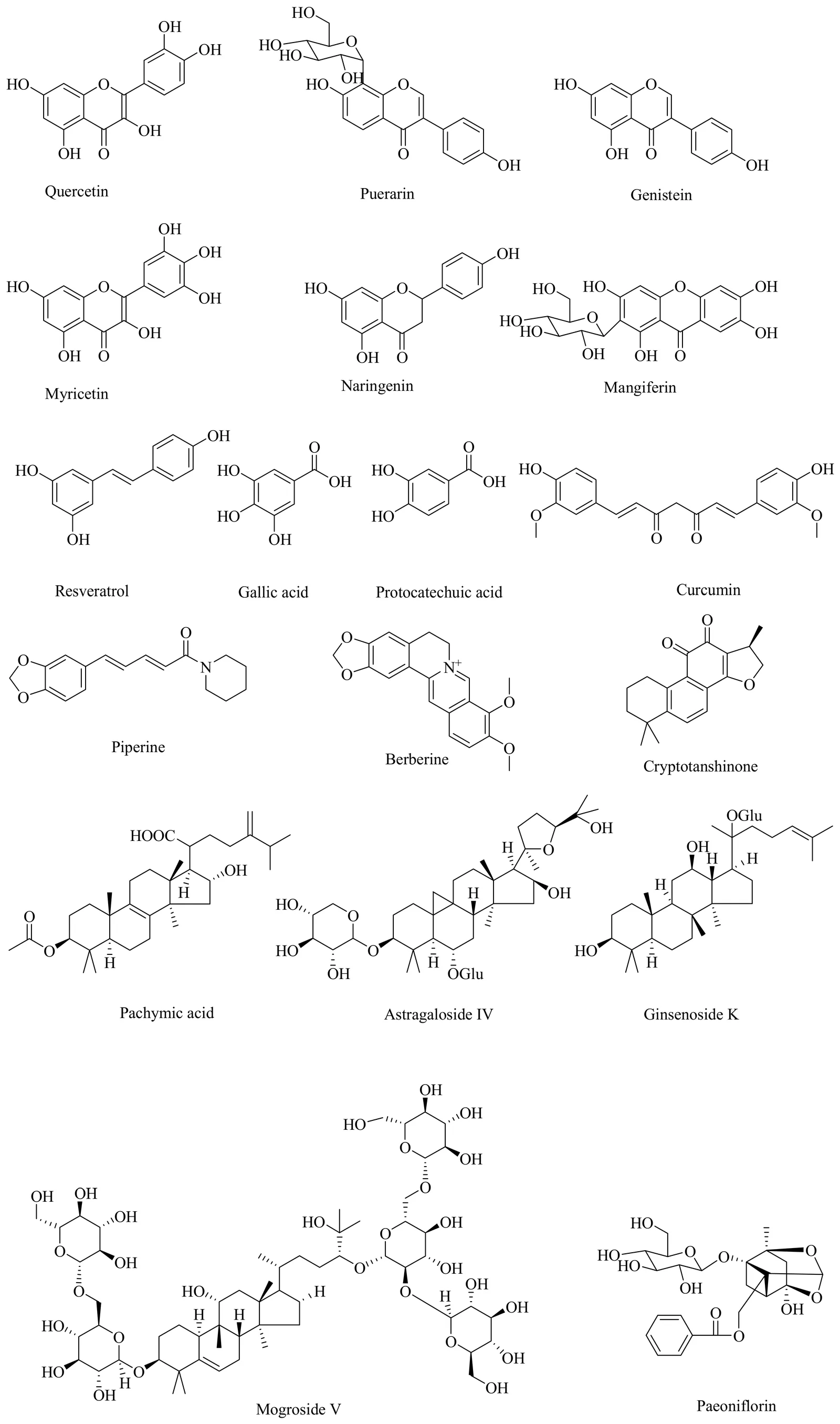
Chemical structures of the bioactive compounds discussed in this review. Only structurally defined single compounds are shown; soybean isoflavones, *Lycium barbarum* polysaccharides (LBPs), and Astragalus polysaccharides (APS) are not included because these terms do not represent a single defined molecular entity.

Complementing the plant-level evidence summarized in Section 3, the following sections present the twenty-one bioactive constituents or constituent classes selected according to the compound-level criteria described in Section 2. The compound-level synthesis was not restricted to constituents of the nine plant-level resources presented in Section 3; accordingly, the representative plant/food sources listed in Table 2 may include species not represented in Section 3. The compounds are organized into flavonoids, other polyphenols, alkaloids, quinones, terpenoids, and polysaccharides (Table 2). Their reported effects and potential mechanisms are discussed in relation to metabolic, inflammatory, and reproductive endocrine pathways relevant to PMOS. To facilitate visual comparison of their chemical features, the chemical structures of the structurally defined single compounds discussed in this section are presented in Figure 1.

### 4.1. Flavonoids

Flavonoids are a major class of plant polyphenols widely distributed in edible plants, including vegetables, fruits, legumes, and tea [43]. In the PMOS-specific studies summarized below, individual flavonoids have been reported to act across insulin and glucose–lipid signaling, redox–inflammatory balance, and reproductive-endocrine pathways. The bioactive constituents discussed below illustrate these potential mechanisms and their relevance to PMOS-related functional-food research.

#### 4.1.1. Quercetin

Quercetin is a flavonoid compound that naturally exists in a variety of fruits and vegetables, including tea, broccoli, apples and grapes [44]. Due to its antioxidant and anti-inflammatory activities, quercetin has been investigated for its potential relevance to PMOS [45]. Animal studies have shown that quercetin regulates the HPO axis of PMOS rats, reducing free testosterone and increasing estradiol levels. Quercetin has also been reported to increase the numbers of primary pre-antral, antral, and preovulatory follicles and corpora lutea, while reducing follicular atresia, thereby restoring ovarian structure and ovulatory function [46]. In addition, the administration of quercetin (100 mg/kg) in PMOS model rats for 30 consecutive days was associated with improved ovarian histology, normalization of the estrous cycle and improvement in the lipid spectrum [47]. Other studies have shown that quercetin improves IR and reduces hyperandrogenism [48]. Given its wide distribution in common foods, quercetin warrants further investigation as a dietary-derived flavonoid for PMOS-related applications; its occurrence in foods should not, however, be taken as evidence of efficacy at habitual dietary intakes.

#### 4.1.2. Soybean Isoflavones

Soybean isoflavones are found abundantly in soybeans, soy milk, tofu and other soy foods and also exist in medicinal plants such as *Pueraria lobata* and *Sophora flavescens*. They are classified as phytoestrogens [49]. Studies have shown that soybean isoflavones improve metabolic dysfunction and support endocrine homeostasis, which may be beneficial to PMOS [50]. In PMOS animal models, soybean isoflavones are reported to reduce body weight and ovarian volume, improve estrous-cycle disorders, reduce T and LH levels, increase E2 and FSH levels, and reduce oxidative stress and inflammation [51]. At the same time, insulin resistance, hepatic lipid accumulation, follicular development and improvement in overall ovarian function are also recorded in the literature [52]. The reproductive–metabolic effects reported in animal studies, together with their established dietary occurrence, support further investigation of soybean isoflavones in PMOS-related functional-food research. However, the PMOS-specific evidence summarized here remains preclinical, and the reported reproductive and metabolic effects require confirmation in human studies before functional-food efficacy can be inferred [51,52].

#### 4.1.3. Puerarin

Puerarin is the main biologically active isoflavone of *Pueraria lobata*, and it is also a marker compound that supports the therapeutic properties of the plant. Studies show that puerarin can improve insulin resistance, regulate glucose–lipid metabolism, and reduce oxidative stress [53]. Animal studies show that puerarin can reduce the body weight, blood sugar and sex-hormone levels of PMOS rats induced by dehydroepiandrosterone (DHEA), especially LH and T. In addition, puerarin has been shown to improve ovarian tissue structure by expanding the granulosa-cell layer and reducing cystic follicles, thus restoring ovarian morphology and function [54]. Another animal study shows that puerarin can reduce blood sugar and insulin in PMOS rats and improve IR [55]. Puerarin thus shows effects at the intersection of glucose homeostasis and reproductive endocrine function, supporting further investigation of this *Pueraria lobata*-derived isoflavone in PMOS-related functional-food research. The available PMOS evidence is derived from rodent models, and differences in induction paradigms and experimental exposure limit direct translation to dietary or clinical use [54,55].

#### 4.1.4. Genistein

Genistein is an isoflavone naturally found in legumes such as soybeans, lentils, peanuts, and mung beans. It shows phytoestrogenic activity and antioxidant properties, which are the basis of its biological effect [56]. A 42-day genistein intervention was reported to improve body weight, lipid profile and insulin resistance in PMOS animal models and reduce ovarian inflammation and tissue damage [57]. In vitro studies show that genistein can reduce the oxidative stress and inflammation of human ovarian granulosa (KGN) cells induced by TNF-α [56]. In addition, genistein can improve glucose metabolism by regulating adiponectin and its signaling pathway [58]. In general, these findings support the further evaluation of genistein as a candidate dietary isoflavone for PMOS-related metabolic and inflammatory abnormalities. Thus, the animal- and cell-based findings provide complementary mechanistic support, but clinical relevance in women with PMOS remains to be established [56,57,58].

#### 4.1.5. Myricetin

Myricetin is widely found in fruits, vegetables, tea and wine. It is reported to have anti-inflammatory, antioxidant and lipid-lowering effects, and is closely related to metabolic disorders [59]. In a DHEA-induced PMOS model, myricetin has been shown to improve reproductive function by increasing the number of corpora lutea, reducing cystic follicles, and restoring sex-hormone levels and estrous cycles. In addition, myricetin enhances insulin sensitivity, thus reducing metabolic abnormalities and ovarian dysfunction [60]. Although myricetin occurs naturally in commonly consumed foods and beverages, the PMOS-specific evidence summarized here is currently limited to preclinical models and does not establish efficacy at habitual dietary exposure [60].

#### 4.1.6. Naringenin

Naringenin is a flavonoid naturally abundant in citrus fruits, including oranges, grapefruits, and lemons. Research shows that naringenin can improve ovarian dysfunction in PMOS rats by regulating serum sex hormones, enhancing intestinal barrier function, reducing intestinal flora disorders and promoting the development of dominant flora [61]. In PMOS rats, oral naringenin (50 mg/kg, 20 days) can reduce the formation of follicular cysts, increase the numbers of corpora lutea and healthy follicles, and normalize circulating androgen levels—the basis of these effects is the concurrent reduction in ovarian oxidative stress and inflammation [62]. Naringenin can also improve glucose and lipid metabolism in adipose tissue and reduce insulin resistance [63]. With its dual action on the gut–ovarian and metabolic–reproductive axes, these findings support further evaluation of naringenin in citrus-based functional-food formulations for PMOS-related applications. At present, however, the evidence comes from preclinical dosing studies, and it is uncertain whether exposure from citrus foods or food-based formulations would be sufficient to reproduce these effects [61,62,63].

#### 4.1.7. Mangiferin

Mangiferin is a C-glucosyl xanthone found primarily in mango (*Mangifera indica*), mangosteen, and the rhizome of *Anemarrhena asphodeloides*. It improves insulin sensitivity, regulates glucose–lipid metabolism, and exerts anti-inflammatory activity [64]. Mangiferin ameliorates glucose and lipid metabolic dysregulation and hormonal imbalance in PMOS rats, while also restoring ovarian function and inhibiting ovarian-cell apoptosis [65]. These effects are primarily attributed to its strong anti-inflammatory and insulin-sensitizing properties [66]. Unlike the flavonoid scaffolds of the other compounds in this section, the C-glucosyl xanthone scaffold of mangiferin provides an additional structural example for PMOS-related functional-food research. However, the PMOS-specific evidence remains preclinical, and neither comparative efficacy nor dietary-dose relevance have yet been established [65,66].

### 4.2. Other Polyphenols

In addition to the above flavonoids, several non-flavonoid polyphenols are also related to the pathophysiology of PMOS. These include the stilbene resveratrol, the phenolic acids gallic acid and protocatechuic acid, and the diarylheptanoid curcumin. Despite their structural diversity, these compounds share strong antioxidant and anti-inflammatory activities while acting on partially overlapping molecular targets [67]. Their distinct chemical scaffolds broaden the structural diversity considered in PMOS-related functional-food research.

#### 4.2.1. Resveratrol

Resveratrol is a stilbene polyphenol with phytoestrogenic activity, which is found in grapes, berries, soybeans and peanuts. It has antioxidant and anti-inflammatory activity [68]. Research shows that a 30-day resveratrol intervention can improve ovarian morphology, correct estrous-cycle abnormalities, and reduce body weight [69]. In addition, dose-dependent administration of resveratrol can restore ovarian tissue structure and increase plasma estradiol and adiponectin levels, thus improving endocrine, metabolic and reproductive dysfunction in a letrozole-induced PMOS model [70]. Although its occurrence in common foods supports interest in food-based research, the PMOS evidence summarized here remains animal-based, and comparable effects at habitual dietary exposure have not been established [69,70].

#### 4.2.2. Curcumin

Curcumin is a natural polyphenol derived mainly from the plants of the Zingiberaceae family. It is a diaryl-heptanoid polyphenol with potent antioxidant, anti-inflammatory, and lipid-modulating activity [71]. In PMOS animal models, a 21-day curcumin treatment significantly reduced serum E2, LH, and T and the LH/FSH ratio, while restoring ovarian morphology and the estrous cycle [72]. Notably, in a 12-week triple-blind trial (*n* = 54 analyzed; 27 per group), curcumin 1000 mg/day (two 500 mg tablets daily) reduced fasting blood glucose compared with placebo (mean difference −6.24 mg/dL; 95% CI −11.73 to −0.76; *p* = 0.027) and improved menstrual-cycle outcomes (*p* = 0.038), whereas testosterone, SHBG, fasting insulin, and serum lipids showed no significant between-group differences [73]. In a separate 12-week double-blind trial (*n* = 60), 500 mg/day of curcumin significantly reduced body weight (−0.8 vs. −0.2 kg; *p* = 0.03), BMI (*p* = 0.03), fasting glucose (β −2.63 mg/dL; *p* = 0.002), insulin and HOMA-IR (both *p* = 0.02), and total cholesterol and LDL-C (both *p* = 0.001) and raised HDL-C (*p* = 0.01), with upregulation of PPAR-γ and LDL-receptor expression [74]. Curcumin is one of the few compounds reviewed here for which human randomized controlled trial data are available; however, the limited number, sample size, and duration of these trials remain insufficient to establish clinical efficacy. Its established dietary use therefore supports further translational evaluation rather than a current clinical recommendation for PMOS.

#### 4.2.3. Gallic Acid

Gallic acid is a natural polyphenol widely found in grapes, pomegranates, tea leaves, and various herbs. Due to its potent antioxidant and anti-inflammatory properties, it may help prevent metabolic disorders [75,76]. In vivo studies have shown that gallic acid reduces serum T, LH, and the LH/FSH ratio, attenuates hyperglycemia and hyperinsulinemia, and suppresses ovarian inflammation; it also raises circulating estrogen and enhances systemic antioxidant defenses, including the activity of endogenous antioxidant enzymes [77]. Other studies show that gallic acid inhibits oxidative DNA damage and lipid peroxidation in ovarian tissue [75]. These results are consistent with a role for reduced oxidative and inflammatory damage in the observed endocrine and metabolic improvements, although the evidence remains preclinical, and the clinical relevance of this mechanism in PMOS is still uncertain [75,76,77].

#### 4.2.4. Protocatechuic Acid

Protocatechuic acid is a natural polyphenol found in almonds, plums, grapes, brown rice, and citrus fruits [78]. It has many biological activities, including anti-inflammatory and antioxidant effects [79]. Both in vivo and in vitro models have shown that protocatechuic acid alleviates hormonal dysregulation in DHEA-induced PMOS models without significant adverse effects. In vitro mechanistic analyses further reveal that protocatechuic acid inhibits aberrant autophagy and apoptosis in PMOS-related ovarian granulosa cells, thereby maintaining ovarian-cell homeostasis [80]. Protocatechuic acid, a widely available dietary polyphenol, has demonstrated potential for protecting ovarian function and modulating endocrine activity in PMOS. The convergence of animal and cell findings provides mechanistic support, but the absence of human evidence limits conclusions regarding translational efficacy [80].

### 4.3. Alkaloids

Alkaloids are a diverse group of naturally occurring nitrogen-containing compounds with a broad range of pharmacological properties [81]. In the present review, piperine and berberine are considered as examples relevant to metabolic and reproductive pathways implicated in PMOS. Their PMOS-specific evidence differs in type and directness, as discussed in the individual subsections below.

#### 4.3.1. Piperine

Piperine is commonly found in Piperaceae plants such as black pepper and long pepper (*Piper longum*) and is a key bioactive ingredient in many spices. Studies show that piperine has biological properties such as anti-inflammatory, antioxidant and immunomodulatory activities [82], suggesting its potential in improving metabolic health, inhibiting chronic inflammation and helping to control weight [83]. An in silico molecular docking study suggested that piperine may interact with PMOS-related molecular targets associated with hyperandrogenism and menstrual irregularity [84]. As this evidence is computational, it is useful mainly for hypothesis generation and does not establish biological efficacy in PMOS. Separately, piperine has demonstrated antioxidant and metabolic effects in non-PMOS experimental models [85], providing a supportive pharmacological context rather than direct PMOS evidence. At present, piperine is therefore better regarded as a compound of mechanistic interest in PMOS research, and its occurrence in culinary spices does not imply that ordinary dietary intake would produce PMOS-related effects.

#### 4.3.2. Berberine

Berberine is an isoquinoline alkaloid, which is abundant in a variety of MFH plants, including *Coptis chinensis* and *Phellodendron amurense* [86]. Its most notable property is its ability to act as a natural activator of AMP-activated protein kinase (AMPK), the same energy-sensing kinase targeted by metformin; AMPK activation regulates glucose–lipid metabolism, increases energy expenditure, improves insulin resistance, and exerts antioxidant activity [87]. In a letrozole-induced PMOS model, berberine normalized circulating hormones, improved insulin resistance, restored ovarian morphology, and reduced granulosa-cell apoptosis [88]. Further studies show that berberine can restore the estrous cycle and ovarian morphology of PMOS rats, while regulating serum hormone levels and endometrial-related gene and protein signaling pathways [89]. A recent study reported that in DHEA-induced PMOS rats, a 4-week berberine intervention restored estrous cycle and insulin sensitivity, reduced serum LH, T, and fasting insulin (FINS) levels, and reduced hyperandrogenism and dyslipidemia, while retaining normal ovarian tissue structure [90]. The available animal studies make berberine relevant to further PMOS research, particularly in relation to metabolic and reproductive dysfunction. Its occurrence in MFH plants does not, however, mean that isolated berberine should be treated simply as a dietary ingredient, and the evidence summarized here remains preclinical rather than evidence of clinical efficacy in women with PMOS [88,89,90].

### 4.4. Quinones

Quinones comprise a structurally diverse class of naturally occurring compounds with multiple physicochemical and biological properties and potential applications in food- and health-related fields [91]. Among the quinone-related compounds considered in this review, the abietane-type diterpene quinone cryptotanshinone provides a representative example relevant to PMOS research.

#### Cryptotanshinone

Cryptotanshinone is a characteristic abietane-type diterpene of *Salvia miltiorrhiza*—an MFH plant used for both medicinal purposes and food coloring—and has also been reported in selected related Lamiaceae species [92]. PMOS research shows that cryptotanshinone can reduce the abnormal apoptosis of ovarian granulosa cells, thus helping to maintain the homeostasis of ovarian cells [93]. Separately, studies in non-PMOS obesity-related models have shown that cryptotanshinone can ameliorate glucose-metabolic abnormalities and insulin resistance [94]; these findings are considered a supportive metabolic background rather than direct PMOS evidence. Cryptotanshinone has also been shown to improve the sex-hormone profile in PMOS and to reduce serum LH, the LH/FSH ratio, testosterone, and the inflammatory mediator TNF-α [95]. Thus, direct PMOS-related evidence supports reproductive and endocrine effects of cryptotanshinone, whereas findings from non-PMOS metabolic models provide a complementary mechanistic context. These data support further investigation of cryptotanshinone as a candidate MFH-derived bioactive compound for PMOS-related applications.

### 4.5. Terpenoids

Terpenoids comprise a structurally diverse group of plant secondary metabolites, encompassing subclasses such as monoterpenes, sesquiterpenes, diterpenes, and triterpenes, and are associated with a broad range of biological activities [96]. The five terpenoid compounds discussed below illustrate this structural diversity and their reported relevance to metabolic, inflammatory, and reproductive-endocrine processes in PMOS.

#### 4.5.1. Pachymic Acid

Pachymic acid is a lanostane-type tetracyclic triterpene, which is highly enriched in the medicinal mushroom *Poria cocos* and also exists in *Ganoderma lucidum*. Both are widely used in broths and decoctions as MFH ingredients. It can regulate inflammation signals and glucose homeostasis [97]. In PMOS animal models, it has been shown that continuous administration of different doses of pachymic acid for 4 weeks can significantly improve insulin resistance and reduce inflammatory markers in a dose-dependent manner [98]. In addition, studies have shown that pachymic acid can inhibit ovarian tissue apoptosis and reduce serum T and LH levels and LH/FSH ratios, thus protecting ovarian structure and function [99]. Pachymic acid therefore provides an example of an edible fungal triterpene relevant to PMOS-related dietary research. The PMOS-specific evidence for pachymic acid is currently confined to rodent studies, and its relevance to dietary exposure or human intervention remains uncertain [98,99].

#### 4.5.2. Mogroside V

Mogroside V is a cucurbitane-type triterpene glycoside isolated from the fruit of *Siraitia grosvenorii*. Its sweetness is about 300 times that of sucrose, and the calorie contribution is negligible. As a calorie-free natural sweetener, it has the biological properties of regulating glucose metabolism and providing antioxidant effects [100]. In vivo studies show that mogroside V can improve the ovarian microenvironment of PMOS rats by increasing the number of corpora lutea and restoring the thickness of the granulosa-cell layer, thus supporting follicular growth and ovulation [101]. The animal findings make mogroside V of interest for PMOS-related research, including future work on food applications. However, the tested preparation and dose differ markedly from ordinary sweetener use, and it is not known whether food-relevant exposure would reproduce the reported ovarian effects [101].

#### 4.5.3. Paeoniflorin

Paeoniflorin is a biologically active monoterpene, which is mainly found in medicinal and food-use plants, such as *Paeonia lactiflora* and *Paeonia suffruticosa*. Reports show that peony petals, pollen, roots and seeds have significant culinary and therapeutic potential [102]. It has been reported that paeoniflorin has anti-inflammatory, antioxidant and immunomodulatory activities [103]. In a PMOS animal model, a 4-week paeoniflorin intervention improved DHEA-induced estrous-cycle disorders, normalized ovarian weight and reproductive-hormone levels, and slowed down the progression of ovarian fibrosis [104]. In terms of mechanism, paeoniflorin reduces ovarian oxidative stress by activating mitophagy, thus supporting granulosa-cell proliferation and follicle growth [105]. Another study found that paeoniflorin improved endometrial receptivity, regulated inflammatory factors, and restored the normal estrous cycle of PMOS rats [106]. This mitophagy-centered mechanism distinguishes paeoniflorin from the other terpenoid compounds discussed in this section and highlights mitochondrial quality control as a mechanistic hypothesis for further PMOS-related research. Although the reported rodent studies provide mechanistically complementary findings, confirmation across standardized preparations and human studies is still lacking [104,105,106].

#### 4.5.4. Astragaloside IV

Astragaloside IV is the main cycloartane-type triterpene saponin of *Astragalus membranaceus*, with antioxidant and anti-inflammatory activities and the ability to regulate glucose metabolism [107]. In vitro mechanism studies show that astragaloside IV regulates the proliferation and apoptosis of human ovarian granulosa (KGN) cells, which is functionally associated with the improvement in ovarian function [108]. In addition, in obese PMOS rats, astragaloside IV reduced LH, testosterone, fasting insulin, and blood sugar, reduced obesity and insulin resistance, and restored ovarian morphology [109]. Further in vivo research has demonstrated that astragaloside IV effectively reduces oxidative stress, normalizes sex-hormone levels, and suppresses ovarian-cell apoptosis in PMOS rats [110]. Together with the Astragalus polysaccharides discussed in Section 4.6.2, these findings illustrate two structurally distinct bioactive classes from the same botanical source; however, their effects have been evaluated in different preclinical systems and should not be interpreted as directly comparable [108,109,110].

#### 4.5.5. Ginsenoside K

Ginsenoside K, commonly referred to in the literature as ginsenoside compound K (CK), is a dammarane-type triterpene glycoside generated by the gut-microbial deglycosylation of major protopanaxadiol-type ginsenosides (e.g., Rb1, Rb2, Rc) found in *Panax ginseng*, *P. quinquefolius*, and *P. notoginseng* [111]. Because most parent ginsenosides are poorly absorbed intact, ginsenoside K, the chief microbially activated metabolite, accounts for much of the systemic bioactivity of orally consumed ginseng and displays markedly higher bioavailability together with anti-diabetic and anti-inflammatory activity [112]. In PMOS model mice, ginsenoside K reduced hyperandrogenism and estrous-cycle disruption, restored steroidogenesis-related enzyme expression, and reduced cystic follicle development [113]. Separately, non-PMOS metabolic studies have reported reductions in fasting glucose and circulating lipids [114], as well as modulation of enterohepatic bile acid circulation and gut-microbiota composition [115]. These latter findings provide metabolic and mechanistic context but are not considered direct PMOS evidence. The reported effects of ginsenoside K on gut microbiota and bile acid metabolism in non-PMOS models suggest a potentially relevant mechanistic pathway for future PMOS research; however, a gut microbiota–bile acid–ovary axis has not yet been directly demonstrated for ginsenoside K in a PMOS-specific model.

### 4.6. Polysaccharides

Polysaccharides are macromolecular substances with high molecular weight, which resist direct absorption by the gastrointestinal tract to a large extent. Therefore, their systemic effect is usually mediated through the intestine—through the prebiotic remodeling of the gut microbiota, the production of short-chain fatty acids by fermentation and the downstream regulation of mucosal immunity—rather than directly entering the circulation [116]. This unique mechanism makes polysaccharides particularly relevant to PMOS, because intestinal flora imbalance in PMOS is increasingly considered a factor leading to insulin resistance, hyperandrogenemia and chronic low-grade inflammation.

#### 4.6.1. *Lycium barbarum* Polysaccharides

*Lycium barbarum* polysaccharides (LBPs) are the main biologically active ingredient of wolfberry fruit. Being derived from a fruit, LBPs have a long-term safe consumption record in the traditional diet. A series of biological activities of LBPs have been described, the most notable of which is regulating glucose metabolism, reducing oxidative stress and alleviating inflammation [117]. In vivo studies have shown that a 28-day LBP intervention in PMOS rats can reduce fasting blood glucose and insulin resistance, while correcting sex-hormone imbalance. Ovarian histology improved, large cystic follicles decreased, and the normal ovarian structure partially recovered [118]. Together with the leaf-based preparations discussed earlier, fruit-derived LBPs illustrate a chemically and anatomically distinct *Lycium barbarum* preparation; however, the PMOS-specific evidence for LBPs remains limited to preclinical animal data [118].

#### 4.6.2. Astragalus Polysaccharides

Astragalus polysaccharides (APSs) are the main biologically active macromolecules in *Astragalus membranaceus*, which play a key role in regulating glucose metabolism and immune homeostasis [119]. In PMOS mouse models, APS administration not only improved insulin resistance and dyslipidemia but also reduced oxidative stress and restored gut-microbiota diversity [120]. In vitro mechanism studies show that APSs inhibit excessive autophagy in ovarian granulosa cells derived from PMOS, thus reducing the harmful effects on ovarian function [121]. Together with astragaloside IV, APSs illustrate structurally distinct bioactive classes from the same botanical source. However, the APS evidence remains preclinical, and its proposed intestinal–immune and granulosa-cell mechanisms require further validation in PMOS-specific translational studies [120,121].

**Table 2:** Chemically defined bioactive compounds from MFH plants with reported PMOS-related effects and comparative evidence profiles, organized by chemical class.

Chemical Class	Compound	Representative Plant/Food Sources	Reported PMOS-Related Effects and Mechanisms	PMOS Model/Study System	Evidence Profile	PMOS-Specific Evidence Reference(s)
Flavonoids	Quercetin	Tea, broccoli, apples, and grapes	Modulation of reproductive-hormone profiles and HPO-axis-related outcomes; restoration of folliculogenesis and ovarian morphology; improvement in insulin resistance and hyperandrogenism	DHEA-induced rat model; letrozole-induced rat model (animal)	A + Massoc	[46,47,48]
	Soybean isoflavones	Soy foods (soybeans, soy milk, tofu); roots of *Pueraria lobata* and *Sophora flavescens*	Normalization of reproductive hormones; suppression of oxidative stress and inflammation; amelioration of IR and hepatic lipid accumulation; restoration of estrous cyclicity and follicular development	Letrozole-induced rat model; DHT-induced PMOS rat model (animal)	A + Massoc	[51,52]
	Puerarin	Root of *Pueraria lobata*	Normalization of LH and testosterone; improvement in insulin resistance; remodeling of ovarian morphology	DHEA-induced rat model; letrozole-induced rat model (animal)	A + Massoc	[54,55]
	Genistein	Legumes (soybeans, lentils, peanuts, and mung beans)	Attenuation of TNF-α–induced oxidative stress and inflammation in granulosa cells; improvement in IR and lipid profile via adiponectin; body weight reduction	DHEA-induced C57BL/6 mouse PMOS model; TNF-α-induced KGN cell model; letrozole-induced rat model; DHEA + hCG + high-fat-diet PMOS-IR rat model (animal + in vitro)	A + C + Mpert	[56,57,58]
	Myricetin	Fruits, vegetables, tea, and wine	Enhancement of insulin sensitivity; restoration of corpus luteum number, sex-hormone profile and estrous cyclicity; improvement in ovarian dysfunction	DHEA-induced C57BL/6J mouse model (animal)	A + Mpert	[60]
	Naringenin	*Citrus fruits* (Citrus spp.)	Reduction in androgen levels; amelioration of glucose–lipid dysregulation; restoration of ovarian function; correction of gut dysbiosis and intestinal barrier dysfunction	Letrozole-induced rat model; DHEA-induced rat model (animal)	A + C + Mpert	[61,62,63]
	Mangiferin	*Mangifera indica* (mango), mangosteen, and *Anemarrhena asphodeloides*	Restoration of hormonal balance; suppression of ovarian-cell apoptosis; insulin-sensitizing and anti-inflammatory action	Letrozole- and high-fat-diet-induced PMOS rat model; DHEA-induced rat model (animal)	A + Massoc	[65,66]
Other polyphenols	Resveratrol	Grapes, berries, soybeans, and peanuts	Restoration of ovarian morphology and estrous cyclicity; reduction in body weight; elevation of circulating estradiol and adiponectin	Letrozole-induced rat model (animal)	A + Massoc	[69,70]
	Curcumin	*Curcuma longa* (turmeric)	Reduction in serum LH, testosterone, and LH/FSH ratio; restoration of ovarian morphology and estrous cyclicity; reduction in fasting blood glucose and improvement in menstrual regularity; improvement in lipid homeostasis	DHEA + HFD-induced mouse model; DHEA-induced rat model; human RCTs (human RCT + animal)	H + A + Massoc	[71,72,73,74]
	Gallic acid	Grapes, pomegranates, and tea leaves	Reduction in serum T, LH, and the LH/FSH ratio; attenuation of hyperglycemia and hyperinsulinemia; suppression of ovarian inflammation; protection against ovarian DNA oxidative damage and lipid peroxidation	Estradiol-valerate-induced polycystic ovary rat phenotype; letrozole-induced mouse model (animal)	A + Massoc	[75,77]
	Protocatechuic acid	Almonds, plums, grapes, brown rice, and citrus fruits	Inhibition of aberrant autophagy and apoptosis in ovarian granulosa cells; correction of hormonal dysregulation; antioxidant action	DHEA-induced PMOS C57BL/6 mouse model; primary granulosa cells isolated from PMOS ovaries (animal + in vitro)	A + C + Mpert	[80]
Alkaloids	Piperine	*Piper nigrum* and *Piper longum*	Predicted interactions with PMOS-related molecular targets associated with hyperandrogenism and menstrual irregularity; hypothesis-generating computational evidence	In silico molecular docking study of PMOS-related targets (computational; hypothesis-generating)	Comp	[84]
	Berberine	*Coptis chinensis* and *Phellodendron amurense*	Normalization of reproductive-hormone profiles and estrous cyclicity; improvement i insulin resistance and glucose–lipid metabolism; restoration of ovarian morphology; reduction in granulosa-cell apoptosis	Letrozole + HFD-exposed rat model; letrozole-induced rat model; DHEA + HFD-induced PMOS-IR rat model (animal)	A + C + Mpert	[88,89,90]
Quinones	Cryptotanshinone	*Salvia miltiorrhiza*	Downregulation of CTBP1-AS in KGN cells; improvement in reproductive-hormone and inflammatory abnormalities; reduction in HMGB1/TLR4/NF-κB-related expression	Human PMOS primary granulosa cells, KGN cells; hCG + insulin-induced rat, granulosa cells (animal + in vitro)	A + C + Massoc	[93,95]
Terpenoids	Pachymic acid	*Poria cocos* and *Ganoderma lucidum* (edible fungi)	Improvement in insulin resistance; suppression of ovarian apoptosis and inflammation; preservation of ovarian function	Letrozole-induced rat model (animal)	A + Mpert	[98,99]
	Mogroside V	Fruit of *Siraitia grosvenorii*	Regulation of glucose metabolism; promotion of follicular development and ovulation	Letrozole combined with high-fat-diet rat model (animal)	A + Massoc	[101]
	Paeoniflorin	*Paeonia lactiflora* and *Paeonia suffruticosa*	Restoration of estrous cyclicity and reproductive hormones; attenuation of ovarian fibrosis and inflammation; improvement in endometrial histology and receptivity-related markers	DHEA-induced rat model; letrozole-induced rat model (animal)	A + Massoc	[104,106]
	Astragaloside IV	*Astragalus membranaceus*	Regulation of granulosa-cell proliferation and apoptosis; reduction in oxidative stress; normalization of sex-hormone levels; improvement in insulin resistance and glucose metabolism; restoration of ovarian morphology	DHEA-induced rat model + KGN system; letrozole + high-fat/high-sugar-diet-induced obese PMOS rat model; letrozole-induced rat model (animal + in vitro)	A + C + Mpert	[108,109,110]
	Ginsenoside K	Gut-microbial metabolite of *Panax ginseng, P. quinquefolius,* and *P. notoginseng*	Reduction in hyperandrogenism and estrous-cycle abnormalities; restoration of steroidogenic enzyme expression; reduction in cystic follicle development	DHEA-induced PMOS rat model (animal)	A + C + Mpert	[113]
Polysaccharides	*Lycium barbarum* polysaccharides (LBPs)	*Lycium barbarum* fruit	Regulation in glucose metabolism and insulin resistance; correction of sex-hormone imbalance; restoration of ovarian histology with reduction in large cystic follicles	Letrozole combined with high-fat-diet rat model (animal)	A + Massoc	[118]
	Astragalus polysaccharides (APS)	Astragalus membranaceus	Improvement in insulin resistance and dyslipidemia; reduction in oxidative stress; rebalancing of aberrant autophagy in granulosa cells; restoration of gut-microbiota diversity	DHEA-induced BALB/C mouse model; testosterone-propionate + C2-ceramide-induced rat granulosa-cell model (animal + in vitro)	A + C + Massoc	[120,121]

Note: “Representative plant/food sources” indicates reported natural sources of the chemically defined compounds and does not necessarily represent the source from which the compound used in the corresponding PMOS study was isolated or obtained. Listing a plant or food source likewise does not imply that the isolated compound has the same food-use or regulatory status as the source material; such status remains ingredient- and jurisdiction-specific. These representative sources are not intended to correspond one-to-one with the nine plant-level resources presented in Section 3. “Reported PMOS-related effects and mechanisms” includes only outcomes supported by the PMOS-specific evidence cited in the final column. Findings derived solely from studies of dietary sources, phytochemistry, general pharmacology, or non-PMOS metabolic, inflammatory, antioxidant, or other mechanistic models are treated as contextual or mechanistic background and are cited only in the relevant narrative sections. “PMOS model/study system” specifies the experimental or clinical context in which the compound was evaluated. Where applicable, the experimental species, cellular system, induction agent, relevant co-induction procedure, and human study design are reported. Different PMOS models reproduce partially distinct endocrine, ovarian, metabolic, inflammatory, and reproductive phenotypes; therefore, findings should be interpreted within the context of the specific model or study system used and should not be assumed to be directly interchangeable across models or extrapolated to clinical efficacy. “PMOS-specific evidence reference(s)” includes studies that directly evaluated the listed compound in a PMOS-related human, animal, or cellular setting, together with PMOS-related computational studies where specifically indicated. Computational studies are not classified as direct biological evidence; where included, they are explicitly identified as in silico, hypothesis-generating evidence and should not be considered equivalent to direct in vivo, in vitro, or human evidence. “Evidence profile” provides a descriptive summary of the PMOS-related evidence available for each resource: H, human-participant clinical/intervention evidence; A, in vivo animal evidence; C, cellular evidence; Mpert, mechanism-focused evidence that includes direct experimental manipulation of a proposed mediator or pathway; Massoc, mechanism-focused molecular, biochemical, signaling, omics, or microbiome evidence without direct causal perturbation; and Comp, computational hypothesis-generating evidence. Where Mpert is assigned, Massoc is not additionally listed. Mpert indicates that at least one mechanism-related experiment involved direct perturbation and does not imply that all proposed mechanisms for that resource have been causally verified. Non-PMOS general pharmacological studies are not counted in the evidence profile. The profile is descriptive and does not constitute a formal GRADE or risk-of-bias assessment. Abbreviations; APS—Astragalus polysaccharides; FSH—follicle-stimulating hormone; HPO—hypothalamic–pituitary–ovarian; IR—insulin resistance; LBPs—Lycium barbarum polysaccharides; LH—luteinizing hormone; LH/FSH—luteinizing hormone to follicle-stimulating hormone ratio; MFH—medicine–food homology; PMOS—polyendocrine metabolic ovarian syndrome; RCT—randomized controlled trial; T—testosterone; TNF-α—tumor necrosis factor-alpha.

### 4.7. Limited Human Evidence and Translational Context

The evidence base reviewed in this article is overwhelmingly preclinical. Among the 58 primary studies included in the evidence synthesis, 55 are animal or in vitro studies, whereas only three human randomized controlled trials are currently available (Table 3). The human studies are therefore presented separately in this section solely to provide limited translational context and should not be interpreted as establishing clinical efficacy. Even in these three trials, sample sizes were small (54–66 participants), intervention durations were relatively short (8–12 weeks), and several prespecified metabolic, hormonal, and secondary outcomes did not show significant between-group differences. The observed benefits were mainly related to metabolic outcomes, including reductions in fasting blood glucose, body weight, BMI, and selected lipid parameters, as well as improvements in menstrual-cycle outcomes in one curcumin trial. In the licorice trial, body weight decreased by 6.24 kg (−7.0%), fasting blood glucose decreased by 26.0%, and lipid parameters improved; however, the supplement was co-administered with a low-calorie diet, making it impossible to fully attribute the observed effects to licorice alone [20]. In the larger curcumin trial, fasting blood glucose and menstrual regularity improved, whereas testosterone, sex-hormone-binding globulin (SHBG), fasting insulin, and lipid parameters showed no significant between-group differences [73]. Overall, these data show that the clinical evidence basis of MFH-derived agents in PMOS is still in the preliminary stage. The mechanistic pathways discussed in the following section should therefore be regarded as biologically plausible and hypothesis-generating, rather than clinically established.

The extent of methodological reporting also differed across the three trials. The licorice trial used random-number-table allocation and a double-blind placebo-controlled design, but allocation concealment and the specific blinded parties were not clearly reported; 66 of 72 randomized participants were included in the final analysis [20]. The 1000 mg/day curcumin trial reported 1:1 block randomization, allocation concealment using consecutively numbered opaque containers prepared by an independent third party, blinding of participants, the researcher, and the analyst, no loss to follow-up, and intention-to-treat analysis; however, the authors noted insufficient statistical power for the testosterone outcome [73]. The 500 mg/day curcumin trial used computer-generated randomization and a matched placebo, but allocation concealment and the specific blinded parties were not described; 50 of 60 randomized participants completed the trial, and the use of different manufacturers for curcumin and placebo was acknowledged as a limitation [74]. Adverse-event reporting was not identified in the published reports of these three trials. Accordingly, the available human findings should be interpreted as preliminary efficacy signals, while the limited sample sizes, short intervention periods, incomplete safety reporting, and study-specific methodological limitations preclude firm conclusions regarding clinical efficacy.

### 4.8. Study-Level Evidence Characteristics

To complement the resource-level synthesis in Table 1 and Table 2, Table 4 summarizes the study-level characteristics and major limitations of the non-RCT evidence, including preclinical animal and cellular studies, human-derived cellular or observational analyses, and computational studies. The three human randomized controlled trials remain summarized separately in Section 4.7 and Table 3. For each study, Table 4 reports the experimental model or study system, intervention dose or experimental exposure, duration, sample size, principal PMOS-related outcomes, and major study limitations. These study-level characteristics also provide the basis for the comparative evidence profiles shown in Table 1 and Table 2.

## 5. Mechanisms Underlying the Effects of Medicinal and Edible Plants on PMOS

**Figure 2:**
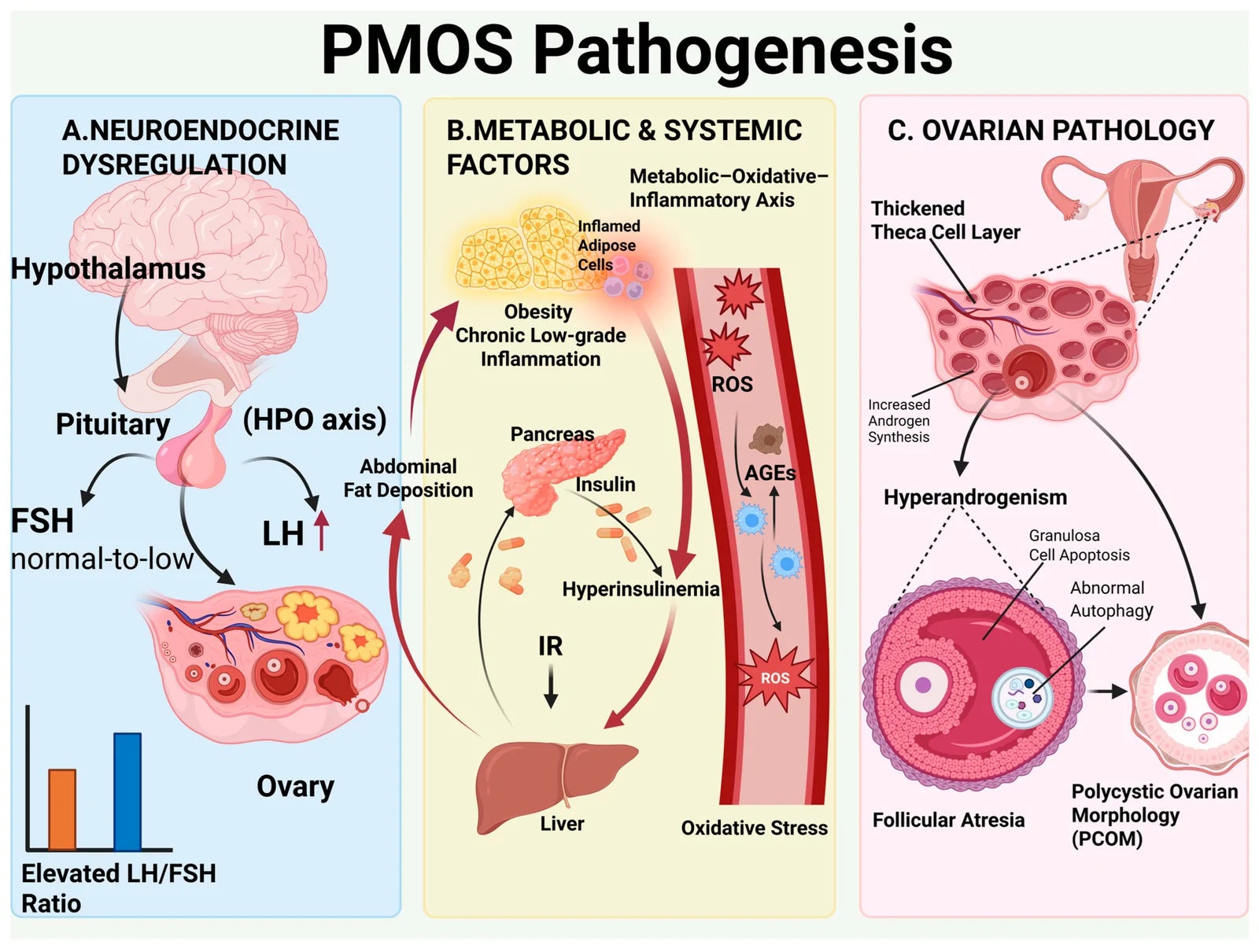
The multifactorial pathogenesis of PMOS. (**A**) Neuroendocrine dysregulation: accelerated GnRH pulsatility within the HPO axis elevates LH and shifts the LH/FSH ratio toward LH dominance. (**B**) Metabolic and systemic factors: obesity-driven adipose inflammation, insulin resistance with compensatory hyperinsulinemia, and accumulation of ROS and AGEs together sustain a self-amplifying metabolic–oxidative–inflammatory loop. (**C**) Ovarian pathology: the convergence of these inputs drives theca-cell hypertrophy and excess androgen biosynthesis (hyperandrogenism), while dysregulated granulosa-cell apoptosis and autophagy promote follicular atresia and the characteristic polycystic ovarian morphology (PCOM). Arrows indicate the direction of regulatory or pathophysiological influences, while upward arrows indicate increases in the corresponding factors. Abbreviations: AGEs, advanced glycation end-products; FSH, follicle-stimulating hormone; HPO, hypothalamic–pituitary–ovarian axis; IR, insulin resistance; LH, luteinizing hormone; PCOM, polycystic ovarian morphology; ROS, reactive oxygen species. Figure created with BioRender.com.

In addition to its clinical heterogeneity, PMOS is best understood as a multi-factorial disease in which neuroendocrine, metabolic and ovarian local disorders reinforce each other. At the central level, the pulse abnormality of gonadotropin-releasing hormone (GnRH) in the HPO axis increases LH secretion and changes the LH/FSH ratio to LH-dominated. At the peripheral level, insulin resistance and compensatory hyperinsulinemia synergize with this increased LH, driving ovarian theca cells to produce excessive androgens, while obesity-related chronic low-grade inflammation and systemic oxidative stress further sustain the self-amplifying metabolic–oxidative–inflammatory cycle. At the ovarian level, these inputs disrupt steroid production, follicular development and granulosa-cell homeostasis—clinically manifested as hyperandrogenemia, follicular atresia, and dysregulated granulosa-cell apoptosis and autophagy (Figure 2).

**Figure 3:**
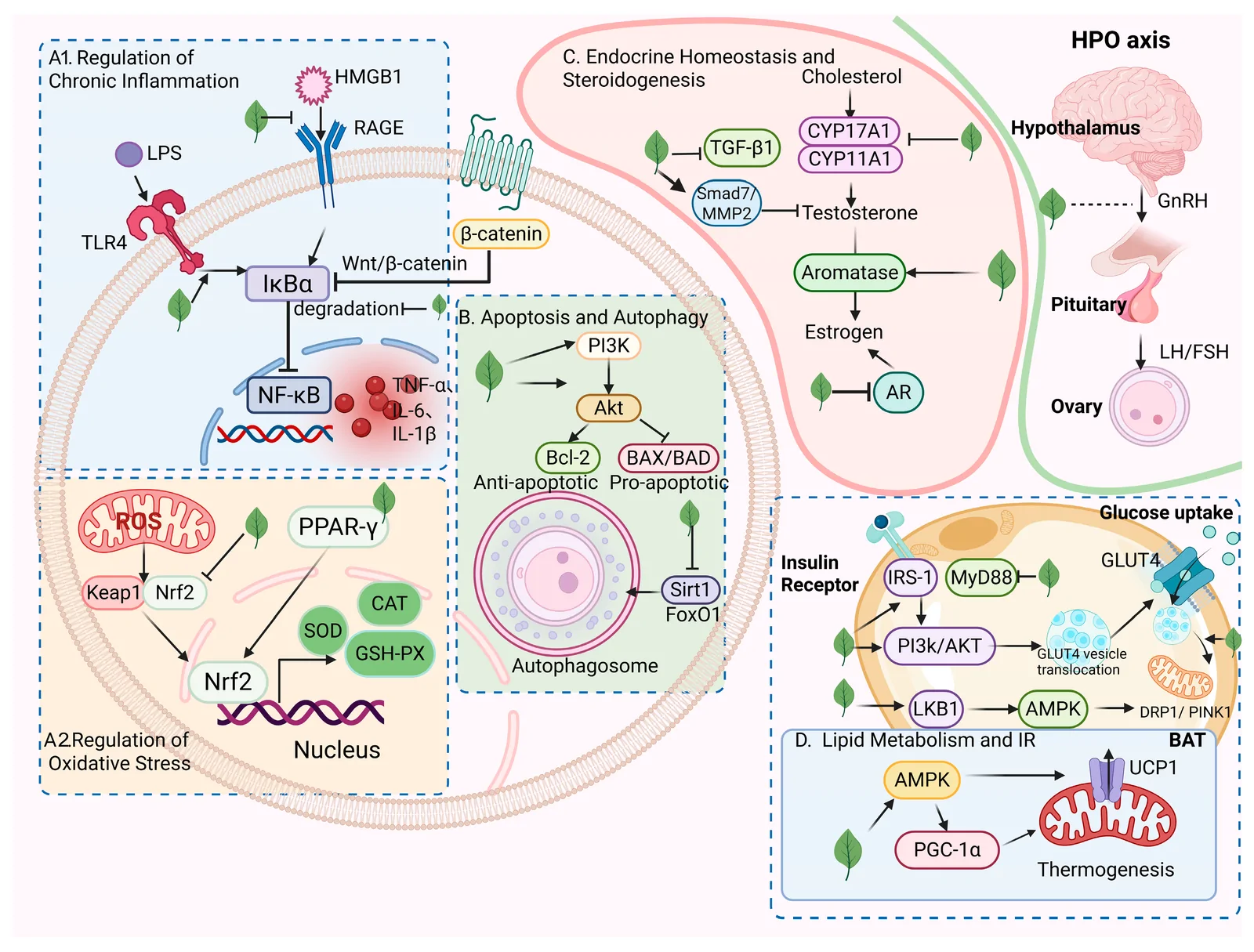
Multi-target regulatory mechanisms of MFH-derived bioactive compounds in PMOS, corresponding to the four pathway-level themes discussed in Section 5.1, Section 5.2, Section 5.3 and Section 5.4. ((**A1**) Inflammation and oxidative stress: suppression of TLR4/NF-κB, HMGB1/RAGE, and Wnt/β-catenin signaling, alongside activation of the Keap1–Nrf2/HO-1 antioxidant axis (with PPAR-γ-mediated anti-inflammatory–antioxidant crosstalk (**A2**)). (**B**) Granulosa-cell apoptosis and autophagy: rebalancing of the PI3K/Akt–Bcl-2/BAX/BAD axis and suppression of the hyperactive SIRT1/FoxO1 autophagy axis. (**C**) Endocrine homeostasis and steroidogenesis: modulation of the HPO axis (GnRH/LH/FSH), suppression of TGF-β1 and the steroidogenic enzymes CYP17A1/CYP11A1, upregulation of aromatase (CYP19A1), and antagonism of the androgen receptor (AR). (**D**) Lipid metabolism and insulin resistance: restoration of IRS-1/PI3K/Akt/GLUT4 insulin signaling (involving MyD88 inhibition), LKB1/AMPK activation with DRP1/PINK1-mediated mitophagy, and AMPK–PGC-1α-driven UCP1 thermogenesis in brown adipose tissue (BAT). Green leaf icons indicate signaling nodes linked to the phytocompounds in the reviewed literature; arrows and blunt-ended lines show the reported direction of change. The evidence supporting individual links varies in strength, ranging from pathway-perturbation experiments to expression-based associations and broader mechanistic context. Abbreviations: (as listed above). Figure created with BioRender.com.

In response to this pathology, MFH-derived compounds do not appear to act on a single node, but rather, they engage multiple nodes: inflammation and oxidative stress, granulosa-cell fate, endocrine and steroidogenic regulation, and insulin signaling. In this review, “multi-target” refers to the broader pattern seen across the evidence base: different compounds and preparations have been reported to affect several, sometimes overlapping, pathways. It does not imply that these compounds act synergistically when combined. The mechanistic support is not uniform across the studies reviewed here. Pathway-perturbation or rescue experiments provide stronger evidence of pathway involvement than changes in pathway-related expression, while findings from non-PMOS or computational studies are used as mechanistic context rather than direct PMOS-specific validation. Since most of the mechanism evidence is still preclinical research, the pathway summarized in Figure 3 should be understood as a convergent mechanism with biological rationality rather than a target that has been clinically verified in humans. Accordingly, Section 5.1, Section 5.2, Section 5.3 and Section 5.4 summarize pathway- and cell-level mechanisms involving inflammatory and redox regulation, granulosa-cell fate, steroidogenesis, and metabolic signaling, whereas Section 5.5 extends the discussion to emerging omics and systems-level evidence and its current limitations.

### 5.1. Regulation of Chronic Inflammation and Oxidative Stress

Chronic low-grade inflammation is widely regarded as a hallmark of PMOS and shows close links with hyperandrogenism, insulin resistance, and obesity. Both clinical and experimental data indicate elevated circulating inflammatory markers in women with PMOS, including TNF-α, IL-6, IL-1β, and IL-18, as well as C-reactive protein [122], and ovarian inflammation is amplified by the accumulation of advanced glycation end-products (AGEs) and upregulation of their receptor RAGE; obesity, insulin resistance, and hyperandrogenism reinforce this state in concert [123]. At the molecular level, activation of the TLR4/NF-κB signaling pathway represents an important mechanism underlying inflammatory responses in PMOS [124]. Modulation of these interconnected inflammatory and metabolic pathways by bioactive compounds derived from functional foods has been increasingly explored as a potential strategy for managing PMOS-related inflammation [125]. Several MFH-derived compounds have been reported to modify these pathways in PMOS-related preclinical studies, although the mechanistic support differs between compounds. For example, soybean isoflavones prevent the activation of NF-κB and inhibit the production of inflammatory mediators by attenuating the phosphorylation, ubiquitination, and degradation of IκBα [51]. In a PMOS rat model, mangiferin reduced inflammatory cytokines alongside changes in NF-κB- and AKT-related signaling [66]. In addition, HMGB1, as a damage-associated molecular pattern (DAMP) protein, promotes the release of inflammatory cytokines by binding to RAGE and activating NF-κB [126]. Pachymic acid inhibits the HMGB1/RAGE axis—a key amplifier of inflammatory signaling—thereby suppressing NF-κB activation [98]. In a separate letrozole study, paeoniflorin reduced endometrial inflammatory markers while increasing Wnt3a/β-catenin expression [106]. This links Wnt/β-catenin-related signaling to the treatment response but does not establish it as the causal pathway.

Because oxidative stress and chronic low-grade inflammation are closely related and reinforce each other to promote the progression of the disease [127], the “metabolic-oxidation-inflammatory axis” has become a key link between reproductive and metabolic disorders in PMOS [128]. Excessive reactive oxygen species (ROS) and the weakening of antioxidant capacity together drive redox imbalance and aggravate ovarian dysfunction [18]. The Nrf2/HO-1 pathway is the main defense mechanism for cells to resist oxidative stress [129]. Recent studies show that functional foods with antioxidant biological activity can activate such defense networks to alleviate metabolic disorders [130]. Angelica extracts have been linked to Keap1/Nrf2/HO-1-related antioxidant responses in PMOS models [42]. The piperine evidence cited for this pathway comes from non-PMOS experimental work [85] and is therefore used here only as general pharmacological context. Curcumin treatment also reduced ovarian oxidative stress while increasing PPAR-γ expression in a DHEA-induced PMOS model [72]. This finding is consistent with PPAR-γ involvement, but does not by itself show that PPAR-γ mediated the broader antioxidant and anti-inflammatory response.

In summary, these findings illustrate how MFH-derived bioactive compounds engage complementary inflammatory (TLR4/NF-κB, HMGB1/RAGE, Wnt/β-catenin) and antioxidant (AMPK–Nrf2/HO-1, PPAR-γ) pathways within the metabolic–oxidative–inflammatory cycle, thereby influencing the reproductive and metabolic features of PMOS pathophysiology.

### 5.2. Modulation of Ovarian Granulosa-Cell Apoptosis and Autophagy

Abnormal folliculogenesis in PMOS is closely associated with accelerated granulosa-cell apoptosis and dysregulated autophagy. Excessive apoptosis exhausts the granulosa-cell group, thus limiting follicular development, which eventually leads to follicular atresia and ovulation failure [131]. Therefore, restoring the homeostasis of granulosa cells is crucial to saving ovarian function [132]. Several MFH-derived compounds target key pathways that govern granulosa-cell homeostasis. Ovarian autophagy dysregulation is typically driven by excessive activation of the SIRT1/FoxO1 signaling axis, whereby SIRT1 overexpression disrupts normal follicular development [133,134]. In the testosterone/ceramide-treated granulosa-cell model, APS reduced autophagosome abundance and LC3-II/LC3-I together with lower Sirt1 and FoxO1 protein expression [121]. Because the pathway itself was not experimentally perturbed, these findings show an association with Sirt1/FoxO1-related autophagy rather than a demonstrated causal inhibition of the pathway. By regulating the Hippo-YAP pathway, pachymic acid reduces phosphorylated YAP and TAZ levels, thus reducing ovarian damage in PMOS rats [99]. The PI3K/Akt cascade is a key regulator of granulosa-cell proliferation and apoptosis. For berberine, PI3K/AKT blockade in granulosa-cell experiments attenuated its effects on proliferation and apoptosis [88], providing more direct support for pathway involvement at the cellular level. At the same time, protocatechuic acid inhibits granulosa-cell apoptosis through the PI3K/Akt/mTOR axis [80].

Taken together, apoptosis and autophagy are recurring mechanistic themes in these studies, but the evidence ranges from pathway-perturbation experiments to changes in pathway-related expression.

### 5.3. Regulation of Endocrine Homeostasis and Steroidogenesis

Hormonal imbalance—usually manifested as a change in the ratio of hyperandrogenemia to estrogen/progesterone—is considered a characteristic pathological feature of PMOS. At the center of this disorder is the HPO axis, in which hypothalamic neurons send signals to the anterior pituitary gland to release LH and FSH; these gonadotropins then act on ovarian granulosa cells and theca cells to drive follicular development [135]. In PMOS, however, aberrant gonadotropin secretion kinetics disrupt gonadal steroidogenesis, leading to elevated mean LH levels and an abnormally high LH/FSH ratio [136]. The intervention studies discussed below involve three main levels of endocrine regulation: upstream signaling, steroidogenic enzymes, and hormone-receptor-related pathways.

First, we discuss upstream cytokine signaling. The overexpression of TGF-β1 drives hyperandrogenemia and plays a key role in the pathophysiology of the disease [137]. In a DHEA-induced PMOS model, paeoniflorin reduced ovarian fibrosis and altered TGF-β1/Smad-related expression [104]. These findings support the involvement of TGF-β1/Smad-related signaling, although the study did not establish it as the sole mechanism underlying the endocrine response.

Next, we discuss steroidogenic enzymes. Several studies have linked PMOS-related interventions to changes in enzymes involved in androgen and estrogen biosynthesis [138,139]. Paeoniflorin treatment was accompanied by lower CYP17A1 and CYP11A1 expression together with improvement in hyperandrogenic features [104]. In the ginsenoside K study, changes in BAT CXCL14 and ovarian steroidogenic enzyme expression occurred alongside improved reproductive features, while exogenous CXCL14 produced similar effects [113]. These findings support a role for CXCL14-related signaling, although they do not establish that CXCL14 is required for the effect of ginsenoside K. Naringenin increased CYP19A1 expression together with improvement in androgen-related and ovarian outcomes [62]. Resveratrol was likewise associated with increased nesfatin-1 in the PMOS model [70]. Reported relationships between nesfatin-1 and reproductive hormones in PMOS [140] provide additional context but do not establish nesfatin-1 as the mediator of the resveratrol response.

Last, receptor-level regulation may provide an additional mechanism for endocrine modulation. In a letrozole-induced PMOS rat model, *Pueraria tuberosa* improved circulating sex-hormone profiles and estrous cyclicity [23]; however, direct estrogen-receptor activation has not yet been demonstrated in a PMOS-specific model. A separate quercetin mechanistic arm showed changes in AR–CNP/NPR2-related signaling, although the dose and administration route differed from those used in the main efficacy experiment [48]. Rubus chingii treatment was associated with improved sex-hormone abnormalities together with lower ovarian TXNIP/NLRP3-related signaling [37].

Taken together, these studies link MFH-derived interventions with changes in upstream signaling, steroidogenic enzymes, and hormone-receptor-related pathways, although the strength of mechanistic evidence differs among them.

### 5.4. Improvement in Lipid Metabolism and Insulin Resistance

Reproductive and endocrine abnormalities in PMOS are aggravated by metabolic dysfunction (especially obesity and insulin resistance). Therefore, the rates of ovulation, conception, pregnancy and live birth are significantly reduced [141]. In addition, obesity actively promotes the production of excessive androgens [142]. At the same time, elevated circulating androgens inhibit the production of uncoupling protein 1 (UCP1) in brown adipocytes, thus impairing mitochondrial respiration and postprandial thermogenesis [143]. This androgen-induced damage weakens BAT activity, ultimately reducing overall energy expenditure and metabolic flexibility [144].

In AMPK and BAT thermogenesis, the AMPK signal is located in the center of the energy steady state: it activates BAT, supports mitochondrial homeostasis, regulates fat generation, and drives the browning of white adipose tissue [145]. Broader studies of MFH resources have linked these pathways to the regulation of lipid metabolism [146]. Within PMOS models, quercetin treatment improved metabolic and reproductive outcomes together with increased ovarian AMPK/SIRT1-related signaling [47]. Myricetin provides more direct evidence for a BAT-related contribution, as BAT-focused experiments, including BAT resection, attenuated the reported metabolic effects [60]. By contrast, the AMPK-related thermogenic effects reported for cryptotanshinone were obtained outside a PMOS-specific model [94] and are therefore considered metabolic context rather than direct PMOS mechanistic evidence.

In insulin signaling, IR is considered an important factor in the pathogenesis of PMOS, which is closely related to hyperandrogenemia and metabolic–reproductive combined disfunction [147]. AKT is a key regulatory factor for cell survival, metabolism and cell cycle control and plays a central role in insulin signaling pathways associated with diabetes [148]. IRS-1 is a key substrate for insulin receptors. IRS-1 activates PI3K and then phosphorylates the Ser473 and Thr308 of downstream AKT [149]. Activated AKT then acts on key targets such as GLUT4 to enhance cellular glucose uptake, thereby helping to stabilize blood glucose [150]. Therefore, it is crucial to restore the PI3K/Akt-mediated insulin signal. Among the PMOS-related studies, mangiferin treatment improved metabolic outcomes together with changes in AKT-related signaling [66], while Angelica sinensis extract was associated with PI3K/Akt-, MAPK4-, and JNK-related changes alongside improved glucose metabolism [39]. Genistein has also been linked to improved metabolic regulation in PMOS-related models [58]. In contrast, the IRS-1/Akt/GLUT4 findings reported for piperine [83], the AMPK-related metabolic effects reported for berberine [151], and the DRP1/PINK1-related skeletal-muscle mitophagy reported for ginsenoside K [114] come from broader metabolic studies rather than direct PMOS-specific pathway experiments. These findings are retained as mechanistic context. Anti-inflammatory signaling may also intersect with glucose regulation [152]. In the glycyrrhizin PMOS study, improved glucose metabolism occurred together with changes in TLR9/MyD88/NF-κB- and Akt/GLUT4-related signaling [153].

Taken together, the metabolic evidence comes from both PMOS-specific studies and broader metabolic pharmacology. Within PMOS models, several interventions were associated with changes in AMPK-, PI3K/Akt-, and inflammation-related signaling. Some of the more detailed metabolic mechanisms, however, have so far been demonstrated only outside PMOS. They remain useful for biological interpretation but should not be treated as PMOS-specific pathway validation.

### 5.5. Omics and Systems-Level Perspectives

Beyond the pathway-level mechanisms described above, a limited but emerging body of research has applied omics and systems-level approaches to MFH-derived interventions in PMOS. Among the intervention studies reviewed, two of the more integrative examples are *Angelica sinensis* and mangiferin. In a letrozole- and high-fat-diet-induced PMOS rat model, *A. sinensis* root aqueous extract was investigated using ovarian RNA sequencing together with 16S rDNA sequencing, identifying treatment-associated changes in ovarian gene expression and gut-microbial composition [39]. Similarly, mangiferin was evaluated in a letrozole- and high-fat-diet-induced PMOS rat model using RNA-seq and 16S rRNA sequencing, together with protein-level validation, revealing changes in pathways related to apoptosis, inflammation, insulin resistance, and gut-microbial composition [65].

Microbiome-focused analyses have also been reported for naringenin [61] and *Lycium barbarum* leaf preparations [35]. In the naringenin study, gut-microbiome alterations were evaluated together with ovarian and intestinal SIRT1/PGC-1α-related responses; transcriptomic data from human oocytes were additionally used for pathway screening, but intervention-specific RNA sequencing was not performed [61]. *Lycium barbarum* leaf intervention was associated with changes in gut-microbial diversity and composition alongside improvements in reproductive hormones, insulin-related parameters, and ovarian morphology in a PMOS mouse model [35]. Collectively, these findings suggest that MFH-derived interventions may influence distributed regulatory processes involving ovarian molecular responses, metabolic signaling, and host–microbiome interactions rather than isolated molecular targets alone.

Related computational and disease-level systems evidence should be interpreted separately. Network pharmacology analysis, molecular docking, and molecular simulation have been used to identify potential PMOS-related targets of piperine, including PPARG, H6PD, NR3C1, FOS, and CYP17A1 [84]. These computational findings are hypothesis-generating and do not establish biological activity or therapeutic efficacy. Separately, an integrated serum metabolomic and proteomic study in women with PMOS identified interconnected abnormalities involving sex-hormone and uric acid metabolism, oxidative stress and NAD-related processes, and arachidonic acid–inflammatory signaling [128]. This disease-level multi-omics study provides a systems framework for PMOS pathophysiology but does not directly validate any MFH-derived intervention.

Taken together, intervention-specific integrated multi-omics evidence for MFH-derived preparations and compounds remains limited. Future studies should combine chemically standardized preparations with transcriptomics, proteomics, metabolomics, lipidomics, microbiome profiling, and pharmacokinetic analyses within the same experimental framework. Single-cell and spatial transcriptomic approaches may further resolve cell-type- and tissue-specific responses in granulosa cells, theca cells, ovarian immune cells, adipose tissue, and other metabolically relevant compartments. Such designs would help distinguish direct molecular actions from secondary systemic responses and provide a stronger mechanistic basis for translational evaluation.

## 6. Safety Considerations, Herb–Drug Interactions, and Translational Challenges

The dietary use of an MFH resource should not, by itself, be taken as evidence that a concentrated preparation or isolated constituent is safe at the doses used experimentally. This distinction is particularly important in PMOS because many potential users are of reproductive age, may be planning pregnancy, and may also be receiving metformin, oral contraceptives, or other metabolic therapies. Safety therefore needs to be considered in relation to the specific ingredient, dose, reproductive context, and concurrent medication. The most obvious example is licorice (*Glycyrrhiza* spp.; Section 3.2): glycyrrhizin and its metabolite glycyrrhetinic acid inhibit the kidney 11β-hydroxy steroid dehydrogenase type 2, producing apparent (pseudo-) mineralocorticoid excess—sodium and water retention, hypokalemia, suppressed renin and aldosterone, and hypertension [154,155]. Licorice-related toxicity is dose-dependent, although individual susceptibility also varies. The latest reviews and clinical reports show that long-term exposure to glycyrrhizin can induce pseudohyperaldosteronism, which is characterized by hypertension, hypokalemia and renin–aldosterone system suppression, especially in susceptible people. Therefore, licorice-containing preparations used for PMOS management should specify the standard of glycyrrhizin content and be used with caution in women with cardiovascular or kidney risk factors [156]. Accordingly, future PMOS-related studies of licorice-containing preparations should explicitly characterize glycyrrhizic acid exposure and include appropriate cardiovascular and renal safety assessment.

Herb–drug interactions are another relevant safety issue because women with PMOS may receive concurrent pharmacotherapy. Berberine has been reported to affect CYP3A4/P-glycoprotein-mediated drug disposition; in kidney-transplant recipients, co-administration increased cyclosporine A exposure, providing a human example of an interaction with potential clinical relevance [157]. Piperine also affected CYP3A4 and P-glycoprotein in experimental studies [158,159]. These findings justify caution, but the evidence cited here does not establish clinically relevant interactions with medications commonly used in PMOS management. Such combinations therefore require direct pharmacokinetic and safety evaluation rather than inference from enzyme or transporter effects alone.

Another consideration involves the phytoestrogens in Section 4—soy isoflavones, genistein, puerarin, and resveratrol—which partially function through estrogen-receptor binding. Phytoestrogens exert context-dependent estrogenic or anti-estrogenic effects through estrogen-receptor signaling pathways. Their biological effects are affected by dosage, developmental stage, endogenous hormone status and intestinal microbial metabolism. In PMOS, this variability is particularly relevant because endocrine and metabolic phenotypes differ among patients, and so do reproductive goals. Current evidence is not sufficient to assume that the same phytoestrogen exposure would have the same benefit–risk balance across these different clinical contexts. Although dietary sources are generally considered safe, high-dose supplementation warrants caution in women undergoing hormonal treatment or planning pregnancy [160]. Ingredient-specific safety information remains uneven across the candidates discussed in Section 3 and Section 4. For many, established dietary safety limits, contraindication or reproductive/pregnancy guidance, and clinically relevant interaction data remain insufficient. Experimental doses should not be treated as established safe intake levels, and the lack of reported adverse events does not demonstrate safety. These gaps should be considered unresolved requirements for functional-food translation rather than evidence of presumed safety.

Beyond these safety considerations, important translational challenges remain in the current evidence base. Most of the evidence supporting PMOS-related biological effects of MFH-derived bioactive compounds comes from induced rodent models, and the experimental doses are often substantially higher than realistic dietary exposure levels. Importantly, the rodent induction paradigms represented in this review should not be treated as directly interchangeable. In representative rat models, letrozole induction produced prominent reproductive abnormalities together with comparatively pronounced metabolic disturbances, whereas testosterone-propionate induction more consistently affected reproductive and pituitary–ovarian features; addition of a high-fat diet further modified the metabolic phenotype of both paradigms [161]. DHEA-induced models are also protocol-sensitive, with the extent of reproductive and metabolic abnormalities varying according to factors such as age, dietary co-exposure, and continuity of DHEA administration [162]. Accordingly, reported effects on reproductive hormones, ovarian morphology, insulin resistance, inflammation, or related outcomes should be interpreted in the context of the phenotype generated by the specific induction protocol, and effect magnitudes should not be assumed to be directly comparable across these models. Comparable challenges in translating food-derived bioactives from mechanistic and preclinical evidence into practical nutraceutical applications have also been highlighted in other chronic metabolic disease contexts [163].

A further translational limitation concerns the composition and standardization of the plant preparations used in experimental studies. Most plant-level studies reviewed here administered extracts, essential oils, enriched fractions, or other concentrated multi-constituent preparations rather than the corresponding whole edible material. The qualitative and quantitative phytochemical composition of these preparations may vary with botanical source, cultivar, cultivation conditions, plant part, extraction solvent and yield, processing procedure, batch, and storage conditions. Even where the same species is named, this variation can limit direct comparison between preparations and make reproducible standardization more difficult. Moreover, the overall effect of a multi-constituent preparation may reflect components acting independently, additively, synergistically, or antagonistically. Most of the studies reviewed here were not designed to distinguish among these possibilities. A multi-target response therefore should not, by itself, be taken as evidence of synergy, and a specific type of component interaction should not be assigned without direct combination testing. Experimental exposure to such preparations may also differ substantially from that achievable through habitual dietary intake. This dose–exposure gap is particularly relevant to animal studies using isolated compounds, enriched fractions, or concentrated preparations, because the tested dose should not be assumed to be achievable through ordinary consumption of the corresponding food. Direct conversion of an animal dose into a food-equivalent human intake is also not justified without considering interspecies dose scaling, food-matrix effects, absorption, metabolism, and bioavailability. Thus, the available preclinical findings should be interpreted as biological activity of the specific preparation and exposure tested, rather than as evidence of comparable effects in habitual dietary intake or equivalent efficacy of the whole plant, a food product derived from that plant, or any individual constituent. Future functional-food studies should therefore quantify realistically achievable food-based exposure and relate it to pharmacokinetic and dose–response data.

For functional-food development, dose achievability is only one component of formulation feasibility, and an effective dietary dose cannot be inferred solely from the dose used in an animal or isolated-compound study. Food-matrix interactions should be evaluated because incorporation into a real food may alter the release, solubilization, or stability of a bioactive during processing and gastrointestinal digestion; thus, the nominal ingredient dose does not necessarily correspond to the fraction available for absorption. Processing and storage should therefore be assessed for the retention of relevant marker compounds and formulation stability, while sensory acceptability should be evaluated at ingredient levels intended to provide meaningful dietary exposure. Bioaccessibility—the fraction released from the food matrix and available for intestinal absorption–should be distinguished from systemic bioavailability. Accordingly, future functional-food formulations should be evaluated for ingredient standardization and achievable dietary dose, together with matrix compatibility, processing/storage stability, sensory feasibility, bioaccessibility, and bioavailability.

A related translational barrier is the limited oral bioavailability of several commonly discussed bioactive compounds, including curcumin, quercetin, resveratrol, berberine, and ginsenosides, which may constrain the translation of experimental exposure into clinically relevant systemic exposure. Human evidence also remains limited and heterogeneous, with small sample sizes, short intervention periods, and inconsistent preparations and doses. Overall, these limitations do not negate the potential value of MFH resources but indicate that the current evidence is better suited to informing research hypotheses than to supporting definitive clinical recommendations.

**Figure 4:**
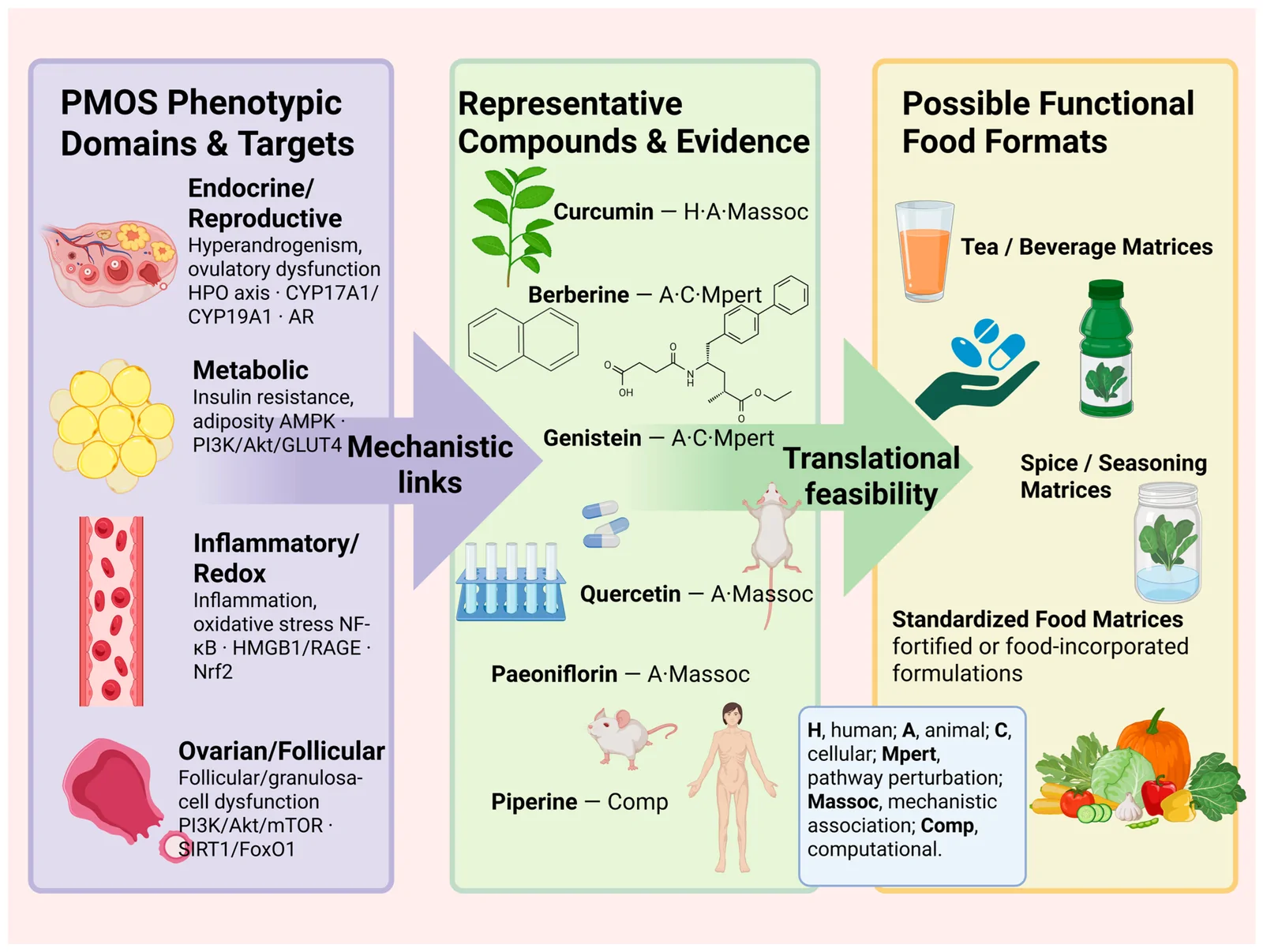
Translational synthesis map linking PMOS-related phenotypic domains, representative molecular targets, selected MFH-derived bioactive compounds, current evidence profiles, and possible future functional-food research formats. The phenotypic domains are overlapping rather than mutually exclusive, and the compounds shown are representative examples. Evidence profiles follow the framework defined in Table 1 and Table 2. The schematic summarizes research relationships and does not indicate phenotype-specific treatment recommendations or established efficacy of the proposed food formats. Figure created with BioRender.com.

Building on the safety, exposure, and herb–drug-interaction considerations discussed above, the translation of MFH-derived interventions should be viewed as a staged pathway rather than as a direct extrapolation from animal models to dietary use. Stage 1 (preparation and food-formulation characterization) should establish botanical identity, cultivar and cultivation conditions where relevant, plant part, preparation and extraction conditions, chemical fingerprints, marker-compound content, batch consistency, the relationship between the experimentally tested dose and exposure realistically achievable through normal food consumption, and formulation-specific food-matrix compatibility, processing/storage stability, and sensory acceptability. Where feasible, candidate formulations should also be validated in prototype or real food products rather than assessed only as isolated extracts or compounds. Stage 2 (translational pharmacology and safety) should define gastrointestinal bioaccessibility, systemic bioavailability, pharmacokinetics, dose translation and tolerable intake, major contraindications, reproductive and pregnancy-related safety, and potential interactions with medications commonly used in PMOS management. Stage 3 (phenotype-informed clinical evaluation) should progress from mechanistically anchored proof-of-concept and dose-ranging studies to adequately powered, longer-duration randomized controlled trials. Participants should be characterized using predefined reproductive/endocrine and metabolic features, including hyperandrogenism, insulin resistance or adiposity, and inflammatory status, with reproductive goals documented alongside relevant mechanistic biomarkers. Clinical outcomes should extend beyond serum hormones and menstrual regularity to include ovulation, fertility-related outcomes where appropriate, glycemic and lipid control, hepatic and cardiovascular risk markers, psychological health, adverse events, and quality of life. Emerging approaches discussed in Section 5.5—including integrated multi-omics, single-cell and spatial analyses, and microbiome profiling—may further help define PMOS subgroups, identify response biomarkers, and clarify host–microbiome–ovarian interactions. The main translational relationships discussed above are summarized in Figure 4, which links PMOS-related phenotypic domains with representative molecular targets, selected bioactive compounds and their current evidence profiles, and possible future functional-food research formats. Until such evidence is available, MFH-derived products should be considered candidates for further investigation as adjunctive nutritional strategies rather than substitutes for established pharmacological and lifestyle management.

## 7. Conclusions

Metformin, ovulation-inducing agents, and hormone therapy remain established options for PMOS management, but their use may be constrained by treatment-specific tolerability, contraindications, adherence, or reproductive considerations. This has contributed to interest in adjunctive dietary strategies that may support long-term metabolic and reproductive management. This review examines the contribution of bioactive compounds derived from MFH plants in this context. Across the nine plant resources and twenty-one compounds presented here, existing preclinical evidence suggests multi-axis biological activity involving the metabolic–oxidative–inflammatory cycle, HPO-axis regulation, ovarian steroidogenesis, and granulosa-cell apoptosis and autophagy. The breadth of these preclinical effects provides a rationale for further investigation of MFH-derived preparations as candidate functional-food ingredients, while not implying equivalence to established pharmacological therapies.

From this synthesis, several priorities for future research emerge. First, MFH-derived sweeteners with reported PMOS-related bioactivity, with mogroside V as a representative example, warrant dedicated investigation to determine whether their metabolic and reproductive effects are maintained at exposures relevant to food use. Second, compounds that influence AMPK-related energy signaling, including berberine, merit mechanistic and comparative studies alongside established metabolic therapies, while recognizing that pathway overlap does not imply therapeutic equivalence. Third, several MFH plants—including *Astragalus membranaceus* and *Lycium barbarum*—provide both small-molecule and macromolecular constituents from the same botanical source. Comparative studies are needed to determine whether whole-plant, fractionated, isolated-compound, or co-formulated preparations differ in efficacy, safety, bioavailability, and reproducibility. Fourth, piperine and emerging formulation approaches such as co-assembly and nanodelivery warrant further investigation for their potential to modify the bioavailability and stability of multi-component preparations, together with careful assessment of pharmacokinetic and herb–drug-interaction risks [164].

There are still important limitations. The vast majority of the primary studies included in this review are preclinical, using heterogeneous experimental systems and rodent induction paradigms, including letrozole, DHEA, testosterone, estradiol valerate, and combined hormonal–metabolic models. Only three human randomized controlled trials are currently available, involving licorice and curcumin. Given their limited number, small sample sizes, and short intervention durations, the available human randomized controlled trials do not provide sufficient evidence to establish clinical efficacy. The heterogeneity of preparation composition, dose, intervention duration, and experimental model further limits direct comparability, and pharmacokinetic data remain scarce—particularly regarding interindividual differences in the gut-microbial activation of compounds such as ginsenoside K. Accordingly, future work should move from standardized preparations, pharmacokinetic and dose-ranging studies, reproductive safety assessment, and validation in real food products toward longer, phenotype-informed randomized trials, with omics and systems-level approaches used to refine mechanistic biomarkers and potential response subgroups. MFH-derived products should therefore be investigated as potential adjunctive nutritional strategies and cannot presently be recommended as substitutes for established pharmacological and lifestyle management.

Integrating traditional MFH dietary practice with contemporary food science, nutritional epidemiology, and reproductive endocrinology offers a path toward developing and evaluating MFH-based functional foods as potential adjuncts to established PMOS management. The compounds and mechanisms documented in this review provide a specific and mechanistically grounded starting point for this translational agenda.

## Figures and Tables

**Table 3:** Summary of human randomized controlled trials of medicine–food homology-derived agents in PMOS.

Agent (Source)	Study Design	Participants (Intervention/Control)	Intervention/Formulation and Dose	Comparator	Duration	Main Clinical Outcomes	Primary Outcome(s)/Trial-Quality and Safety Considerations	Ref.
Licorice (*Glycyrrhiza glabra* extract)	RCT, double-blind, placebo-controlled	66 analyzed (33/33); 72 enrolled	Licorice extract 1.5 g/day + low-calorie diet	Placebo + low-calorie diet	8 weeks	Body weight decreased by 6.24 kg (−7.0%), BMI decreased by 1.64 kg/m^2^, and body-fat percentage decreased by 2.45 percentage points. Fasting blood glucose decreased by 26.0%. Lipid profile improved: TG −37.2%, TC −23.5%, LDL-C −33.0%, and HDL-C +12.2% (between-group *p* < 0.05).	Primary outcome hierarchy not clearly prespecified; adverse-event reporting NR. Random-number-table allocation and double blinding were reported, but allocation concealment and blinded parties were not specified. Of 72 randomized participants, 66 were analyzed. Both groups received a low-calorie diet, limiting interpretation of licorice as a stand-alone intervention.	[20]
Curcumin (*Curcuma longa*)	RCT, triple-blind, placebo-controlled	54 analyzed (27/27)	Curcumin 1000 mg/day (2 × 500 mg)	Placebo	12 weeks	Between-group reduction in fasting blood glucose (MD −6.24 mg/dL; 95% CI −11.73 to −0.76; *p* = 0.027). Menstrual-cycle abnormalities were reduced (amenorrhea: 11.1% vs. 29.7%; oligomenorrhea: 37.0% vs. 51.9%; *p* = 0.038). No significant between-group differences in testosterone, SHBG, fasting insulin, or serum lipids.	Primary outcomes: metabolic indices and androgen level; adverse-event reporting NR. Block randomization, allocation concealment, and blinding of participants, researcher, and analyst were reported; all 54 randomized participants were analyzed by intention to treat. The authors reported <80% power for the testosterone outcome.	[73]
Curcumin (*Curcuma longa*)	RCT, double-blind, placebo-controlled	50 completed/analyzed (24 curcumin/26 placebo)	Curcumin 500 mg/day	Placebo	12 weeks	Compared with placebo, curcumin reduced body weight (−0.8 vs. −0.2 kg; *p* = 0.03), BMI (*p* = 0.03), fasting glucose (β −2.63 mg/dL; 95% CI −4.21 to −1.05; *p* = 0.002), insulin and HOMA-IR (both *p* = 0.02), total cholesterol and LDL-C (both *p* = 0.001), and increased HDL-C (*p* = 0.01).	Primary outcomes: HOMA-IR and insulin; adverse-event reporting NR. Computer-generated randomization and a matched placebo were reported, but allocation concealment and specific blinded parties were not described. Of 60 randomized participants, 50 completed the trial and were analyzed.	[74]

Abbreviations: BMI—body mass index; HDL-C—high-density lipoprotein cholesterol; LDL-C—low-density lipoprotein cholesterol; PMOS—polyendocrine metabolic ovarian syndrome; RCT—randomized controlled trial; SHBG—sex-hormone-binding globulin; TC—total cholesterol; TG—triglycerides. “Triple-blind”—blinding of participants, investigators, and outcome assessors. Participant numbers refer to those analyzed in the primary analysis; enrolled numbers are provided where dropout was reported. The licorice trial co-administered a low-calorie diet in both arms, which limits attribution of the observed effects to licorice alone. The licorice extract was reported to contain 7.03% glycyrrhizic acid (reported as 36.5 mg per 500 mg capsule). Trial-quality and risk-of-bias considerations are qualitative and based on information reported in the published articles; they do not constitute a formal Cochrane RoB 2 assessment. NR, not reported. Lack of adverse-event reporting should not be interpreted as evidence that no adverse events occurred.

**Table 4:** Study-level characteristics and major limitations of the non-RCT PMOS evidence supporting the plant- and compound-level synthesis in Table 1 and Table 2.

Intervention/Agent	Experimental Model/Study System	Dose or Exposure/Duration/Sample Size	Principal PMOS-Related Outcomes	Major Limitation(s)	Ref.
**Panel A. Plant/preparation-level studies corresponding to Table 1**					
*Foeniculum vulgare* essential oil	Estradiol-valerate-induced PMOS in female Wistar rats; single EV 4 mg/kg i.m.; 60 d induction period	FEO 200 mg/kg i.p.; 14 d; n = 5/group (35 total, 7 groups)	Improved steroid-hormone profile and ovarian histology; reduced ovarian cyst number; MDA unchanged	Animal study only; single induction model	[17]
*Licorice hydroalcoholic* extract	Estradiol-valerate-induced PMOS in female NMRI mice	100 or 150 mg/kg by gavage; 3 wk; n = 8/group (32 total)	Improved ovarian morphology and reproductive competence; reduced cystic/atretic follicles	Animal study only	[21]
*Pueraria tuberosa* tuber ethanolic extract	Letrozole-induced PMOS in female Wistar rats	100, 200, or 400 mg/kg; 14 d; n = 6/group (36 total)	Improved sex-hormone profile and ovarian histomorphology; reduced cystic follicles	Animal study only; single induction model	[23]
Total flavonoids from *Eucommia ulmoides leaves* (TFEL)	Letrozole + high-fat-diet-induced PMOS-IR rat model; HFD for 49 d and letrozole 1 mg/kg/day for 28 d	TFEL 55, 110, or 220 mg/kg/day; 21 d; n = NR in abstract	Improved sex-hormone profile and insulin resistance; reduced body/ovarian indices and ameliorated ovarian and pancreatic histopathology	Animal study only; combined hormonal–metabolic induction model	[26]
	DHEA-induced PMOS in female SD rats; DHEA 60 mg/kg s.c. for 21 d	TFEL 50, 100, or 200 mg/kg by gavage; 21 d; n = 10/group (60 analyzed)	Improved reproductive-hormone profile and ovarian/uterine indices; low-dose effect on insulin was non-significant	Animal study only; single induction model	[27]
*Crocus sativus petal* extract (SPE) and petal anthocyanins (SPA)	Testosterone-enanthate-induced PMOS in female mice	SPA 20, 40, or 80 mg/kg; SPE dose levels reported as 50, 100, and 600 mg/kg in a study-level secondary extraction; 14 d; n = 12/subgroup (96 total reported)	Improved reproductive-hormone profile and ovarian endocrine regulation; reduced inflammatory signaling and enhanced antioxidant responses	Animal study only; testosterone-based single induction paradigm; petal-derived preparations differ from the conventionally consumed saffron stigma	[30]
Turmeric *(Curcuma longa)* extract	Letrozole-induced PMOS in female Swiss albino mice; letrozole 6 mg/kg p.o. for 21 d	Turmeric extract 175 mg/kg p.o.; 30 d; n = 6/group (24 total)	Improved reproductive-hormone and glucose–lipid profiles; reduced oxidative/inflammatory stress; increased circulating adiponectin	Animal study only; single turmeric dose	[32]
*Lycium barbarum* leaf aqueous extract	Letrozole + high-fat-diet-induced PMOS in female C57BL/6J mice; letrozole 1 mg/kg/day, continued during treatment	880 or 1760 mg/kg/day by gavage; 29 d; n = 10/group (50 total)	Improved insulin resistance, reproductive-hormone profile, ovarian morphology/estrous cyclicity, and gut-microbiota dysbiosis	Animal study only; combined hormonal–metabolic model	[35]
*Rubus chingii* decoction	DHEA-induced PMOS-IR in female SD rats; DHEA 60 mg/kg s.c. for 20 d and continued during treatment	Decoction 10 mL/kg/day (20 g/200 mL); 4 wk; n = NR/group (64 PMOS -IR rats qualified)	Improved insulin sensitivity, hormone profile, and ovarian morphology; suppressed TXNIP/NLRP3 signaling	Animal study only; DHEA exposure continued during treatment	[37]
*Angelica sinensis* root aqueous extract (WEA)	Letrozole + high-fat-diet-induced PMOS in female SD rats; letrozole 1 mg/kg by gavage with HFD	WEA 2, 4, or 8 g/kg by gavage; 4 wk; n = 10/group; RNA-seq n = 3/group	Improved estrous cyclicity, ovarian histology, hormone profile, insulin resistance and dyslipidemia; altered ovarian signaling and gut microbiota	Animal study only; combined hormonal–metabolic model; RNA-seq performed on a small high-dose subset	[39]
	Letrozole + high-fat-diet-induced PMOS in female SD rats; letrozole 1 mg/kg for 4 wk	WEA 2, 4, or 8 g/kg by gavage; 4 wk; n = 10/group	Improved ovarian histology, reproductive-hormone profile and glucose–lipid metabolism; increased PI3K/AKT phosphorylation	Animal study only; combined hormonal–metabolic model	[40]
	Letrozole-induced PMOS in female SD rats; letrozole 1 mg/kg for 28 d	WEA 2, 4, or 8 g/kg by gavage; 28 d; n = 10/group (60 total)	Improved ovarian histology and hormone profile; reduced inflammatory/oxidative-stress abnormalities	Animal study only; WEA was co-administered during PMOS induction	[41]
*Angelica sinensis* root ethanol extract (EEA)	Letrozole + high-fat-diet-induced PMOS in female SD rats; letrozole 1 mg/kg for 4 wk	EEA 200, 400, or 800 mg/kg by gavage; 4 wk; n = 10/group	Improved estrous cyclicity, ovarian histology, hormone profile and dyslipidemia; attenuated oxidative-stress responses	Animal study only; combined hormonal–metabolic model	[42]
**Panel B. Compound-level studies corresponding to Table 2**					
Quercetin	DHEA-induced PMOS in female rats	25 mg/kg p.o.; 28 d; n = 7/group (42 total)	Reduced hyperandrogenism and improved HPO-axis function, folliculogenesis, and ovarian morphology	Animal study only; single quercetin dose	[46]
	Letrozole-induced PMOS in female Wistar rats; letrozole 1 mg/kg p.o. for 21 d	100 mg/kg by gavage; 30 d; n = 6/group (18 total)	Improved estrous cyclicity, reproductive-hormone and lipid profiles, and insulin resistance; increased ovarian AMPK/SIRT1 signaling	Animal study only; single quercetin dose	[47]
	DHEA-induced PMOS in female SD rats; DHEA 60 mg/kg s.c. for 20 d	Main arm: 100 mg/kg by gavage; 28 d; n = 30/group (3 × 10). Additional mechanistic arm: 4 mg/kg i.p.; duration/n not clearly reported in the provided text.	Improved ovulatory function and ovarian morphology; modulated reproductive hormones, inflammatory/apoptotic markers, and AR–CNP/NPR2 signaling	Animal study only; mechanistic arm used a different quercetin dose and route from the main efficacy arm	[48]
Soybean isoflavones	Letrozole-induced PMOS in female SD rats; letrozole 1 mg/kg p.o. for 21 d	100 mg/kg p.o.; 28 d; n = 5/group (20 total)	Improved estrous cyclicity, ovarian morphology and reproductive-hormone profile; reduced ovarian oxidative stress and inflammation	Animal study only; single isoflavone dose	[51]
Soy isoflavones (NovaSoya preparation)	DHT-induced PMOS in female SD rats; continuous DHT release 83 μg/day via s.c. implant	0.5 or 1 g ISF/kg diet; 8 wk; n = 8/group in cohort 1 and 6/group in cohorts 2–4 (156 total)	Improved insulin sensitivity and lipid profile; reduced body-weight gain and hepatic lipid accumulation; partially normalized follicular development	Animal study only; outcomes were assessed across different cohorts and the ISF preparation contained substantial non-isoflavone constituents	[52]
Puerarin + metformin	DHEA-induced PMOS in female SD rats; 20 d induction	Puerarin 100 mg/kg i.p. + metformin 270 mg/kg by gavage; 4 wk; n = 20/group (80 analyzed, including normal control)	Improved glucose homeostasis and insulin resistance, reproductive-hormone profile and ovarian morphology; reduced inflammatory markers and TLR4/NF-κB-related expression	Animal study only; puerarin was evaluated only in combination with metformin, limiting attribution to puerarin alone	[54]
Puerarin	Letrozole-induced PMOS in female Wistar rats; letrozole 1 mg/kg/day p.o. for 21 d	50, 75, or 100 mg/kg s.c.; 21 d; n = 13/group (78 total)	Improved glucose homeostasis, insulin resistance and ovarian histology; increased PI3K/AKT/FoxO1 phosphorylation	Animal study only; single letrozole-induced model	[55]
Genistein	DHEA-induced PMOS in female C57BL/6 mice; TNF-α-treated KGN cells	5, 10, or 20 mg/kg in vivo; duration NR in supplied text; n = 6/group in vivo; n = 3/group in vitro	Improved ovarian morphology and reproductive-hormone profile; reduced oxidative/inflammatory abnormalities; SAA1 overexpression attenuated genistein-associated effects	Preclinical animal/cell evidence only; no human validation	[56]
	Letrozole-induced PMOS in adult female SD rats; letrozole 1 mg/kg for 21 d	20 mg/kg; 42 d; n = 10/group (40 total)	Improved insulin resistance and ovarian histopathology; reduced TNF-α and MDA and increased SOD; fasting insulin change was not significant	Animal study only; single genistein dose	[57]
	DHEA + hCG + high-fat-diet-induced PMOS-IR in female SD rats; 42 d induction	10, 20, or 30 mg/kg/day; 21 d; n = 8/group in intervention phase	Improved glucose metabolism and insulin resistance; modulated APN/APPL1- and RBP4/PEPCK-related metabolic signaling	Animal study only; combined hormonal–metabolic model; study focused primarily on metabolic outcomes	[58]
Myricetin	DHEA-induced PMOS in female C57BL/6J mice; DHEA 60 mg/kg s.c. for 20 d and continued during treatment	Myricetin 100 mg/kg by gavage; 3 wk; endpoint-specific n = 3–10/group	Improved estrous cyclicity/fertility, hyperandrogenism, ovarian morphology and insulin sensitivity; increased BAT activity	Animal study only; continued DHEA exposure during treatment; endpoint-specific small sample sizes	[60]
Naringenin	Letrozole-induced PMOS in female SD rats; letrozole 1 mg/kg/day p.o. for 28 d; human-oocyte transcriptomic data used for pathway screening	20 mg/kg/day p.o.; 8 wk; n = 6/group (24 rats in final four groups)	Improved reproductive-hormone profile, insulin resistance and ovarian histology; modulated gut microbiota and SIRT1/PGC-1α-related responses	Animal intervention only; human transcriptomic component was limited and not intervention-specific	[61]
	Letrozole-induced PMOS in female SD rats; letrozole 1 mg/kg/day p.o. for 21 d and continued during treatment; human granulosa cells	50 mg/kg/day p.o.; 20 d in Methods; n = 5–6/group; hGC exposure: 2.5 μM	Reduced androgen levels and follicular cysts and improved ovulation-related ovarian features; restored steroidogenesis-related protein expression	Preclinical animal/cell evidence; letrozole continued during treatment	[62]
	DHEA-induced PMOS in female SD rats; DHEA 60 mg/kg s.c. for 20 d; PA-induced IR in differentiated SW872 adipocytes	50 mg/kg/day by gavage; 28 d; n = 10/group (50 total); cells: 8 or 16 μM, 24 h pretreatment	Improved glucose–lipid metabolism and insulin resistance and reduced lipid deposition; effects were attenuated by PKGIα inhibition	Preclinical animal/cell evidence; study focused predominantly on metabolic outcomes	[63]
Mangiferin	Letrozole + high-fat-diet-induced PMOS in female SD rats; HFD for 8 wk, with letrozole 1 mg/kg/day during weeks 5–8	50 or 200 mg/kg/day; ~4 wk after model establishment; n = 6/group (30 total)	Improved glucose–lipid metabolism, insulin resistance, hormone profile and ovarian morphology; reduced ovarian apoptosis and altered gut microbiota	Animal study only; combined hormonal–metabolic induction model	[65]
	DHEA-induced PMOS in female Wistar rats; DHEA 6 mg/kg/day s.c. for 28 d	10, 20, or 30 mg/kg orally; 4 wk; n = 10/group (40 PMOS rats allocated to treatment/model groups)	Reduced glucose/insulin abnormalities and inflammatory cytokines; decreased ovarian weight indices; altered NF-κB- and AKT-related signaling	Animal study only; study focused predominantly on metabolic and inflammatory outcomes	[66]
Resveratrol	Letrozole-induced PMOS in female Wistar rats; oral letrozole 400 or 800 μg/day for 60 d; unilateral ovariectomy performed before treatment for model confirmation	20 mg/kg/day (n = 6) or 30 mg/kg/day (n = 8); 30 d; saline comparator n = 4	Dose-dependent improvement in ovarian morphology and estrous cyclicity; reduced body weight; no significant change in sRAGE	Animal study only; small/unequal groups; unilateral ovariectomy before treatment	[69]
	Letrozole-induced PMOS in female SD rats; letrozole 1 mg/kg orally; induction duration NR in the reported methods	40, 80, or 160 mg/kg; 30 consecutive days; n = 10/group (50 total)	Increased circulating estradiol and adiponectin and improved ovarian morphology; increased ovarian nesfatin-1 and aromatase expression	Animal study only; letrozole-induction duration not specified; Western blotting was limited to the high-dose and control groups	[70]
Curcumin	DHEA + high-fat-diet-induced PMOS in female C57BL/6 mice; 21 d modeling period	Curcumin 0.1 mg/mouse/day alone; combination: curcumin 0.1 mg/mouse/day + astaxanthin 1.6 mg/mouse/day; treatment route/duration relative to modeling NR in supplied text; n = 10/group (50 total)	The combination improved estrous cyclicity, ovarian histology, reproductive-hormone and lipid-related outcomes, oxidative/inflammatory markers, and ovulation-related endpoints, with greater effects than either monotherapy	Animal study only; combined hormonal–dietary model; curcumin dose reported per mouse rather than per kg; combination effects cannot be attributed to curcumin alone, and greater effects than either monotherapy do not by themselves establish synergy	[71]
	DHEA-induced PMOS in female SD rats; DHEA 60 mg/kg/day s.c. for 21 d and continued during treatment	50 mg/kg/day by gavage; 21 d; n = 6/group (18 total)	Improved insulin resistance, ovarian morphology and estrous cyclicity; reduced reproductive-hormone abnormalities and ovarian oxidative stress; increased ovarian PPAR-γ expression	Animal study only; single curcumin dose; DHEA exposure continued during treatment	[72]
Gallic acid	Estradiol-valerate-induced polycystic ovary phenotype in female Wistar rats; single EV 4 mg/kg i.m.; 60-d induction	50 or 100 mg/kg/day orally; 24 d; n = 8/group reported (32 total)	Reduced ovarian inflammatory and oxidative-damage markers and increased antioxidant enzyme activities	Animal study only; Methods ambiguously describe removal of one rat/group for histological model confirmation	[75]
	Letrozole-induced PMOS in female Parkes-strain mice; letrozole 6 mg/kg by gavage for 21 d	75 mg/kg/day orally; 60 d; n = 6/group (18 total)	Improved endocrine–metabolic, ovarian and oxidative/inflammatory abnormalities; increased ovarian AdipoR1 and steroidogenesis/folliculogenesis-related mRNA expression	Animal study only; single gallic acid dose; mechanistic inference based mainly on gene-expression changes	[77]
Protocatechuic acid	DHEA-induced PMOS in female C57BL/6 mice; DHEA 60 mg/kg/day s.c. for 21 d; primary ovarian granulosa cells isolated from PMOS mice	10, 20, or 40 mg/kg i.p.; 8 d; n = 6/group in vivo (42 total); in vitro n = NR	Improved PMOS-related ovarian/hormonal abnormalities; reduced granulosa-cell ROS, autophagy and apoptosis; PI3K/AKT/mTOR-related signaling was implicated	Preclinical animal/cell evidence only; 8 d intervention; in vitro replicate number not reported in supplied methods	[80]
Piperine	Network pharmacology, molecular docking and molecular-dynamics simulations against PMOS-related human protein targets	No biological dosing; 100 ns molecular-dynamics simulations; n = N/A	Predicted interactions with five shortlisted PMOS-related targets, with favorable docking to H6PD and PPARG and supportive simulation stability	Computational evidence only; no cellular, animal or clinical validation	[84]
Berberine	Letrozole + HFD-exposed PMOS model in female SD rats; letrozole 1 mg/kg/day by gavage for 23 d and HFD for 30 d; rat ovarian granulosa cells	95 or 190 mg/kg by gavage; 4 wk; n = 10/group in vivo (50 rats); additional drug-serum donors n = 10/dose; granulosa-cell donor n = NR	Improved insulin-resistance and reproductive-hormone abnormalities, ovarian morphology and apoptosis; PI3K/AKT blockade attenuated Ber-associated effects on granulosa-cell proliferation and apoptosis	Preclinical animal/cell study; pathway blockade was performed in granulosa-cell experiments rather than as in vivo mechanistic validation	[88]
	Letrozole-induced PMOS in female SD rats; letrozole 1 mg/kg by gavage for 21 d	Berberine dose/route not reliably extractable because of an internal drug-name inconsistency in the treatment description; treatment period 21 d; n = 29/group (87 total)	Reduced body weight and estrous-cycle disturbance; altered serum testosterone, estradiol and progesterone levels; improved ovarian morphology; altered endometrial ERα and AKT3/RAC1/PTEN/KRAS-related expression	Animal study only; berberine dosing information is internally inconsistent in the source, limiting reproducibility and interpretation of the intervention	[89]
	DHEA + high-fat-diet-induced PMOS-IR in female SD rats; DHEA 60 mg/kg/day s.c. + HFD for 49 d	40.5, 81, or 162 mg/kg by gavage; 28 d; initial n = 60 for PMOS-IR modeling and n = 12 controls; final n/group after treatment allocation NR	Improved estrous cyclicity; reduced FINS/HOMA-IR, LH and testosterone; improved lipid-related indices and ovarian histology	Animal study only; combined hormonal–metabolic induction model; final treatment-group sample sizes were not explicitly reported in the supplied methods	[90]
Cryptotanshinone	Primary ovarian granulosa cells from women with PMOS and healthy controls; human KGN granulosa tumor cells	KGN cells: 2.5, 5, or 10 μM for 24 h; human donors n = 60/group; cell experiments repeated three times	CTBP1-AS was elevated in PMOS granulosa cells; cryptotanshinone reduced CTBP1-AS expression in KGN cells	Observational human-cell and in vitro evidence only; no animal or clinical intervention; cryptotanshinone effect was assessed mainly at the CTBP1-AS-expression level	[93]
	hCG + insulin-induced PMOS in female SD rats; hCG 3 IU/day with escalating insulin for 22 d; dexamethasone-induced IR in rat ovarian granulosa cells	27 mg/kg orally once daily for 3 wk; n = 12/group (60 rats); in vitro CRY 300 nM; additional granulosa-cell donors n = 6	Reduced body/ovarian weight and reproductive-hormone/inflammatory abnormalities; decreased HMGB1/TLR4/NF-κB-related expression in ovarian tissue and granulosa cells	Preclinical animal/cell evidence only; specific hCG–insulin induction paradigm; pathway findings do not establish clinical efficacy	[95]
Pachymic acid	Letrozole-induced PMOS in female SD rats; letrozole 1 mg/kg by gavage for 21 d	8.33 or 33.32 mg/kg by gavage; 4 wk; n = 18/group overall; ovarian coefficient, histology and molecular analyses each used n = 6/group	Improved insulin resistance, reproductive-hormone abnormalities and ovarian pathology; reduced ovarian inflammatory and HMGB1/RAGE/NF-κB-related markers; rHMGB1 reversed the high-dose-associated improvements	Animal study only; single letrozole model; ovarian histological/molecular endpoints used n = 6/group	[98]
	Letrozole-induced PMOS in female SD rats; letrozole 1 mg/kg by gavage for 21 d	10 or 40 mg/kg by gavage; 4 wk; n = 10/group; histology/TUNEL n = 5/group and Western blot n = 5/group	Improved insulin resistance, estrous-cycle/ovarian abnormalities and reproductive-hormone profile; reduced ovarian apoptosis; PY-60 attenuated the ovarian protective effect	Animal study only; single letrozole model; histological/apoptosis and protein analyses used separate n = 5/group subsets	[99]
Mogroside V preparation (50.42% mogroside V)	Letrozole + high-fat-diet-induced PMOS in female SD rats; letrozole 1 mg/kg/day orally + HFD for 30 d	600 mg/kg/day by gavage; 30 d; n = 10/group (30 total); RNA-seq n reported inconsistently as 4/group for library construction and 3 biological replicates/group for differential-expression analysis	Improved estrous cyclicity, testosterone level and ovarian follicular development; increased glycolysis-related metabolites and ovarian LDHA/HK2/PKM2 expression	Animal study only; combined hormonal–dietary model; intervention was a 50.42% mogroside V preparation rather than purified compound	[101]
Paeoniflorin	DHEA-induced PMOS in female SD rats; DHEA 60 mg/kg/day s.c. for 35 d	20, 40, or 80 mg/kg/day by gavage; 4 wk; n = 10/group for control, PMOS and each PAE group; 8 of the 48 DHEA-treated rats were not accounted for in the reported treatment allocation	Improved estrous-cycle and reproductive-hormone abnormalities and reduced ovarian fibrosis; altered TGF-β1/Smads-related expression	Animal study only; single DHEA model	[104]
	Letrozole-induced PMOS in female SD rats; letrozole 0.5 mg/kg by gavage for 21 d	50 or 100 mg/kg by gavage; 21 d; n = 10/group (50 total)	Improved reproductive-hormone abnormalities and endometrial histology/receptivity-related markers; reduced endometrial inflammatory markers and increased Wnt3a/β-catenin expression	Animal study only; single letrozole model; endometrial receptivity was inferred from surrogate histological/molecular markers rather than implantation or pregnancy outcomes	[106]
Astragaloside IV (AS-IV)	DHEA-induced PMOS in female SD rats; DHEA 6 mg/kg/day s.c. for 20 d; human KGN granulosa tumor cells	AS-IV 20, 40, or 80 mg/kg/day for 20 d, concurrently with DHEA; administration route not specified; n = 30 total, with 18 rats across the three AS-IV groups; KGN: 20, 40, or 80 μg/mL for 48 h; cell experiments ≥ 3 independent repeats	Improved ovarian pathology and hormone abnormalities; increased autophagy- and PPARγ-related responses; inhibitor experiments supported involvement of PPARγ/autophagy in KGN-cell proliferation and apoptosis	Preclinical animal/cell evidence only; AS-IV was co-administered during PMOS induction; per-dose animal n and AS-IV injection route were not explicitly reported	[108]
	Letrozole + high-fat/high-sugar-diet-induced obese PMOS in female SD rats; letrozole 1 mg/kg/day with HFD and 50 g/L glucose water for 30 d	25 or 50 mg/kg by gavage; 21 d; n = 8/group (40 total); treatment started on day 29 of the 30 d induction protocol	Improved insulin resistance, glucose–lipid and reproductive-hormone abnormalities, and ovarian morphology; reduced MAPK/ERK-related phosphorylation and VEGF expression	Animal study only; combined hormonal–metabolic model	[109]
	Letrozole-induced PMOS in female SD rats; letrozole 1 mg/kg by gavage for 21 d	12.5 or 50 mg/kg by gavage; 21 d; n = 12/group (60 total)	The 50 mg/kg dose improved estrous cyclicity, ovarian morphology and hormone abnormalities and reduced oxidative-stress/apoptosis-related changes; the 12.5 mg/kg dose showed no significant improvement	Animal study only; single letrozole model; effects were largely confined to the high-dose group; StAR-related mechanism was expression-based	[110]
Ginsenoside K (CK)	DHEA-induced PMOS in female SD rats; DHEA 60 mg/kg/day s.c. for an initial 20 d and continued throughout the experiment; additional BAT-related experiments in UCP1-luciferase and Db/Db mice and primary brown adipocytes	CK 20 mg/kg/day by gavage for 20 d in rats; PMOS group n not explicitly reported in the supplied methods (59 rats used overall across rat experiments); auxiliary mice: 10 UCP1-luciferase and 10 Db/Db mice	Improved hyperandrogenism, estrous cyclicity, ovarian steroidogenic enzyme expression and cystic follicle abnormalities; CK increased CXCL14, while exogenous CXCL14 produced similar improvements in DHEA- PMOS rats	Preclinical animal/cell evidence only	[113]
*Lycium barbarum* polysaccharides (LBPs; purity ≥90%)	Letrozole + high-fat-diet-induced PMOS-IR in female SD rats; letrozole 1 mg/kg/day by gavage + HFD for 21 d	25, 50, or 100 mg/kg/day by gavage; 28 d; n = 12/group (72 total)	Reduced FBG, FINS and HOMA-IR; improved reproductive-hormone profile and ovarian histopathology; increased ovarian LKB1 and AMPK mRNA/protein expression	Animal study only; combined hormonal–metabolic model; LKB1/AMPK involvement was inferred from total expression changes without phosphorylation/activity or pathway-perturbation analysis	[118]
Astragalus polysaccharides (APS)	DHEA-induced PMOS in female BALB/C mice; DHEA 60 mg/kg/day s.c. for 21 d	APS 400 mg/kg/day by gavage; 28 d; n = 10/group reported (30 mice assigned; 40 mice reported purchased)	Improved insulin resistance, oxidative-stress and lipid abnormalities; altered reproductive/metabolic indices and gut-microbiota composition/diversity	Animal study only; single APS dose; microbiota–metabolic associations were correlational	[120]
Astragalus polysaccharide (APS; purity 90%)	Primary ovarian granulosa cells isolated from normal female SD rats; testosterone-propionate (10^−5^ mol/L) + C2-ceramide (10 μM) for 24 h to induce an autophagy model	APS 100, 200, or 400 μg/mL for 24 h; granulosa-cell donors n = 3 rats	Increased granulosa-cell proliferation and reduced autophagosome abundance and LC3-II/LC3-I; decreased Sirt1 and FoxO1 protein expression	In vitro evidence only; artificial testosterone/ceramide-induced autophagy model rather than an intact PMOS animal model; only three donor rats; no pathway perturbation establishing Sirt1/FoxO1 causality	[121]

**Note:** Study-level information was extracted from the corresponding primary reports. The three human randomized controlled trials are summarized separately in Table 3 and are not duplicated here. NR, not reported in the source article; N/A, not applicable. Where a study contained multiple experimental components, the relevant conditions are reported within the same row or separately where necessary. “Major limitation(s)” refers to concise design- or interpretation-related limitations identifiable from the reported study characteristics and does not represent a formal risk-of-bias assessment.

## Data Availability

No new data were created or analyzed in this study. Data sharing is not applicable to this article.

## Abbreviations

The following abbreviations are used in this manuscript:

AGE	Advanced glycation end-product
AKT	Protein kinase B (AKT serine/threonine kinase)
AMPK	AMP-activated protein kinase
APS	Astragalus polysaccharide(s)
AR	Androgen receptor
BAD	Bcl-2-associated death promoter
BAT	Brown adipose tissue
BAX	Bcl-2-associated X protein
Bcl-2	B-cell lymphoma 2
BMI	Body mass index
CAT	Catalase
CIDEA	Cell death-inducing DFFA-like effector a
CXCL14	C-X-C motif chemokine ligand 14
CYP11A1	Cytochrome P450 family 11 subfamily A member 1
CYP17A1	Cytochrome P450 family 17 subfamily A member 1
CYP19A1	Cytochrome P450 family 19 subfamily A member 1 (aromatase)
DAMP	Damage-associated molecular pattern
DHEA	Dehydroepiandrosterone
DRP1	Dynamin-related protein 1
E2	Estradiol (17β-estradiol)
ERα	Estrogen receptor alpha
ERβ	Estrogen receptor beta
*Esr1*	Estrogen receptor 1 gene
FBG	Fasting blood glucose
FINS	Fasting insulin
FoxO1	Forkhead box O1
FSH	Follicle-stimulating hormone
*Fshr*	Follicle-stimulating hormone receptor gene
GLUT4	Glucose transporter type 4
GPx	Glutathione peroxidase
GST	Glutathione S-transferase
HMGB1	High mobility group box 1
HO-1	Heme oxygenase 1
HPO	Hypothalamic-pituitary-ovarian (axis)
IκBα	Inhibitor of nuclear factor kappa B alpha
IL-1β	Interleukin 1 beta
IL-6	Interleukin 6
IL-18	Interleukin 18
INS	Insulin
IR	Insulin resistance
IRS-1	Insulin receptor substrate 1
JNK	c-Jun N-terminal kinase
Keap1	Kelch-like ECH-associated protein 1
KGN	Human ovarian granulosa-like tumor cell line
LBPs	Lycium barbarum polysaccharides
LH	Luteinizing hormone
*Lhr*	Luteinizing hormone receptor gene
LKB1	Liver kinase B1
MAPK4	Mitogen-activated protein kinase 4
MFH	Medicine–food homology
MMP2	Matrix metalloproteinase 2
mTOR	Mechanistic target of rapamycin
MyD88	Myeloid differentiation primary response 88
NF-κB	Nuclear factor kappa B
NLRP3	NLR family pyrin domain containing 3
Nrf2	Nuclear factor erythroid 2-related factor 2
P	Progesterone
PMOS	Polyendocrine metabolic ovarian syndrome
PCOS	Former name of PMOS; retained in historical source titles and database search strings
PGC-1α	Peroxisome proliferator-activated receptor gamma coactivator 1-alpha
*Pgr*	Progesterone receptor gene
PI3K	Phosphatidylinositol 3-kinase
PINK1	PTEN-induced kinase 1
PPAR-γ	Peroxisome proliferator-activated receptor gamma
Prdm16	PR domain zinc finger protein 16
RAGE	Receptor for advanced glycation end-products
RCT	Randomized controlled trial
ROS	Reactive oxygen species
SIRT1	Sirtuin 1 (silent information regulator 1)
Smad7	SMAD family member 7
SOD	Superoxide dismutase
T	Testosterone
TAZ	Transcriptional coactivator with PDZ-binding motif (WWTR1)
TCM	Traditional Chinese medicine
TGF-β1	Transforming growth factor beta 1
TLR4	Toll-like receptor 4
TLR9	Toll-like receptor 9
TNF-α	Tumor necrosis factor alpha
TXNIP	Thioredoxin-interacting protein
UCP1	Uncoupling protein 1
YAP	Yes-associated protein
